# Reverse prenylation in plants by non‐canonical aromatic prenyltransferases

**DOI:** 10.1111/tpj.70268

**Published:** 2025-06-17

**Authors:** Lukas Ernst, Hesham M.B. Sayed, Ahmed Hassanin, Rebekka Moegenburg, Tomke Meents, Hui Lyu, David Kaufholdt, Mehdi D. Davari, Ludger Beerhues, Benye Liu, Islam El‐Awaad

**Affiliations:** ^1^ Institute of Pharmaceutical Biology Technische Universität Braunschweig Mendelssohnstraße 1 38106 Braunschweig Germany; ^2^ Center of Pharmaceutical Engineering (PVZ) Technische Universität Braunschweig Franz‐Liszt‐Straße 35 A, 38106 Braunschweig Germany; ^3^ Department of Pharmacognosy, Faculty of Pharmacy Assiut University Assiut 71526 Egypt; ^4^ Department of Bioorganic Chemistry Leibniz Institute of Plant Biochemistry Weinberg 3 06120 Halle (Saale) Germany; ^5^ NMR/Biosynthesis Group Max Planck Institute for Chemical Ecology Jena 07745 Germany; ^6^ Institute of Plant Biology Technische Universität Braunschweig Humboldtstraße 1 38106 Braunschweig Germany

**Keywords:** aromatic prenyltransferase, reverse prenylation, prenyltransferase regioselectivity, biotechnological production of xanthones, *Hypericum perforatum*, *Hypericum sampsonii*

## Abstract

Reverse‐prenylated phenolic compounds are an abundant class of bioactive plant natural products. Hyperixanthone A, an inhibitor of multidrug‐resistant *Staphylococcus aureus*, is a polyprenylated xanthone carrying two forward geminal and one reverse prenyl group. Although prenyltransferases responsible for the forward prenylations were identified, the final reverse prenylation reaction remained elusive. No plant enzyme catalyzing reverse prenylation of an aromatic carbon has been described so far. Here we use metabolic profiling and transcriptomic information from *Hypericum perforatum* and *H. sampsonii* to identify homologous enzymes involved in the formation of reverse‐prenylated xanthones and characterize their functions using *in vitro*, *in vivo*, and *in silico* approaches. The identified enzymes are non‐canonical UbiA‐type prenyltransferases, which surprisingly catalyze both forward and reverse prenylations with different regioselectivities. Reconstruction of the enzyme cascade in *Saccharomyces cerevisiae* and *Nicotiana benthamiana* confirmed reverse‐prenylated hyperixanthone A as the major product. Molecular modeling and docking simulations supported by site‐directed mutagenesis suggest two distinct binding modes, which enable forward and reverse prenylations and provide a rationale for the preferred catalysis of the reverse prenyl transfer reaction. The identification of reverse prenylation augments the repertoire of reactions catalyzed by membrane‐bound UbiA‐type plant aromatic prenyltransferases. The insights also provide a new tool for the biotechnological modification of pharmaceutically valuable natural products.

## Significance Statement

Aromatic prenyltransferases (aPTs) are key catalysts in plant specialized metabolism, enhancing bioactivity by attaching hydrophobic prenyl groups to aromatic compounds. While no plant aPT catalyzing reverse prenylation of aromatic carbons has been identified so far, this study reports plant UbiA‐type aPTs from *Hypericum* species, which catalyze reverse prenylation and form the antimicrobial hyperixanthone A *in vitro* and *in vivo*. The results expand our understanding of plant aPTs and offer new tools for biotechnological production of valuable natural products.

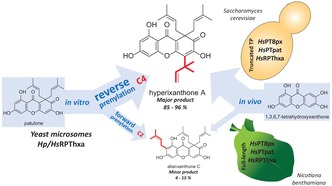

## INTRODUCTION

Plants produce a variety of prenylated phenolic compounds. Their biosynthesis involves the decoration of shikimate and polyketide pathway products (prenyl acceptors) with dimethylallyl, geranyl, or farnesyl units available as corresponding prenyl diphosphates (prenyl donors). The insertion of prenyl units boosts the hydrophobicity and membrane affinity of phenolic specialized metabolites (Mori, 2020; Yazaki et al., 2009). Consequently, the mixed pathway products exhibit a wide array of interesting biological and pharmacological activities (Alhassan et al., 2014; Chang et al., 2021; Yazaki et al., 2009). The underlying prenylation reactions are catalyzed by a group of enzymes known as aromatic prenyltransferases (aPTs). In plants, aPTs involved in the biosynthesis of prenylated phenolic specialized metabolites exclusively belong to the membrane‐integrated enzymes of the UbiA superfamily, which feature up to nine transmembrane helices and two conserved aspartate‐rich motifs. They exhibit narrow acceptor and donor specificities and strict divalent metal ion dependency (de Bruijn et al., 2020; Stec & Li, 2012). Unlike plants, specialized metabolism in microbes involves soluble aPTs, which can be subdivided into dimethylallyltryptophan synthase (DMATS) and αββα fold (ABBA) type enzymes. Microbial soluble aPTs show relatively promiscuous acceptor and donor specificities, the majority not requiring metal ions for catalysis (Chen & Abe, 2021; Chen et al., 2016).

Enzymatic prenylation can occur in either forward (also referred to as normal or regular) or reverse mode (Figure S1). Forward prenylation involves the attachment of C‐1 of a prenyl donor to the prenyl acceptor, whereas attachment of C‐3 results in reverse prenylation. Microbial aPTs catalyzing both forward and reverse prenylations were identified and characterized (Chen & Abe, 2021; Tanner, 2015). For instance, reverse prenylation of fumigaclavine A by the action of FgaPT1 represents a crucial step in the biosynthesis of the ergot alkaloid fumigaclavine C in *Aspergillus fumigatus* (Unsöld & Li, 2006). Furthermore, CymD is a reverse aPT engaged in the biosynthesis of cyclic peptides, such as the antimicrobial cyclomarazine A in the marine actinobacterium *Salinispora arenicola* (Figure S1) (Schultz et al., 2008). Although several plant‐derived aPTs catalyzing forward prenylations are known, only one PT from bakuchi (*Psoralea corylifolia*) has recently been found to catalyze reverse geranylation of a nonaromatic carbon in the biosynthesis of bakuchiol (Zheng et al., 2024). No plant UbiA‐type aPT catalyzing reverse prenylation of an aromatic carbon has been identified to date.

More than 4000 prenylated phenolic compounds, including approximately 240 with reverse prenyl groups on aromatic carbons, have been identified from the plant kingdom (Munakata & Yazaki, 2024). Reverse‐prenylated phenolic compounds display interesting pharmacological activities (Figure S1). A prominent example is licochalcone A, a chalcone from the rhizomes of liquorice (*Glycyrrhiza inflata*) and other *Glycyrrhiza* species. It exhibits well‐established antimalarial, anti‐*Helicobacter pylori* and anti‐cancer activities (Chen et al., 1994; Fukai et al., 2002; Li et al., 2022). Owing to its anti‐inflammatory properties, licochalcone A is used in topical pharmaceutical preparations (Kolbe et al., 2006). Other examples include clausenidin, a reverse‐prenylated coumarin from pink lime‐berry (*Clausena excavate*) with promising antiviral activity (Su et al., 2009), and broussonol A, a flavonol from *Broussonetia kazinoki* showing cytotoxic properties (Zhang et al., 2001). Reverse‐prenylated phenylpyrone, phloroglucinol and xanthone derivatives are predominantly formed by members of the Hypericaceae, Clusiaceae, and Moraceae (Fobofou et al., 2016; Niu et al., 2012; Yoon et al., 2016; Zhang et al., 2019). Plants belonging to the genus *Hypericum* (Hypericaceae) stand out as an abundant source of reverse‐prenylated xanthones, rendering them suitable systems for studying UbiA‐type aPTs that catalyze reverse prenylation of aromatic carbons.

**Figure 1:**
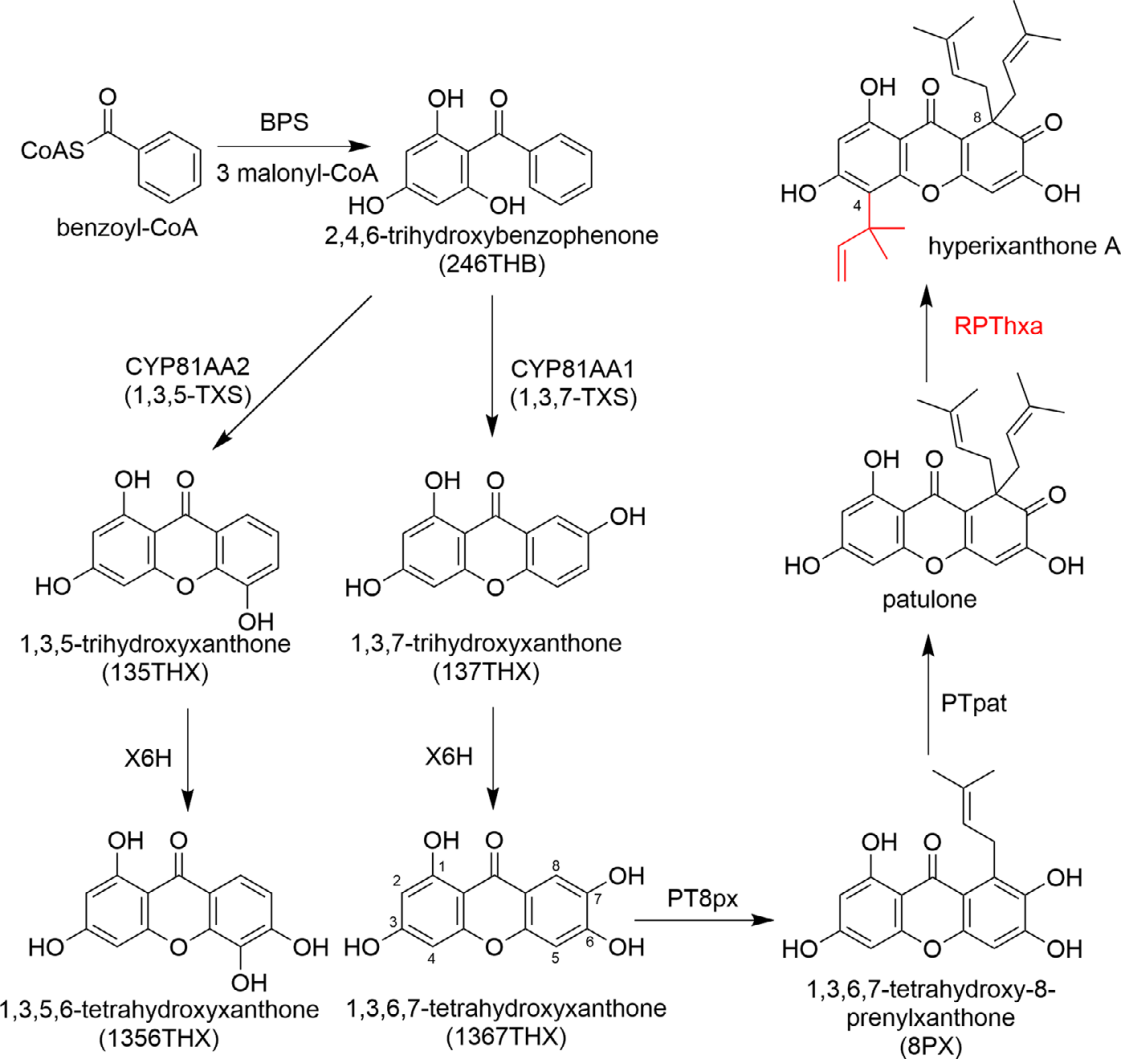
Proposed biosynthetic pathway of hyperixanthone A in *Hypericum* species, the reverse PT studied here (RPThxa) being highlighted in red. BPS, benzophenone synthase. PT, prenyltransferase. TXS, trihydroxyxanthone synthase. X6H, xanthone 6‐hydroxylase; PT8px, 1,3,6,7‐tetrahydroxyxanthone 8‐PT; PTpat, 1,3,6,7‐tetrahydroxy‐8‐prenylxanthone 8‐PT; RPThxa, patulone 4‐reverse PT.

The genus *Hypericum* comprises approximately 500 species distributed worldwide. The successful use of the flagship plant St. John's wort (*Hypericum perforatum*) as antidepressant has encouraged the exploration of further *Hypericum* species for active metabolites (Zhao et al., 2015). *Hypericum sampsonii* is traditionally used in Chinese folk medicine as anti‐inflammatory agent for the treatment of burns, dermatitis and arthritis (Chen et al., 2020). The plant is a rich source of polyprenylated phloroglucinols and xanthones (Lin & Wu, 2003; Sun et al., 2023; Xiao et al., 2008). An example of a reverse‐prenylated xanthone is hyperixanthone A (Figure 1), which was isolated from *H. sampsonii* and exhibits potent activity against multidrug‐resistant *Staphylococcus aureus* (MRSA) (Xiao et al., 2008). Hyperixanthone A is a representative of a large group of reverse‐prenylated xanthones, which occur in fungi and plants and show diverse pharmacological activities (Figure S2b). Interestingly, the introduction of a reverse prenyl group at C‐4 of tri‐ and tetrahydroxyxanthones markedly increased the antimalarial activity of constituents from *Garcinia vieillardii* (Hay et al., 2004). Moreover, gerontoxanthone I from *Cudrania cochinchinensis* showed pronounced activity against vancomycin‐resistant bacteria (Fukai et al., 2005) (Figure S2b).

Key enzymes responsible for the biosynthesis of polyprenylated xanthones in *Hypericum* sp. were identified (Figure 1). The biosynthesis involves the formation of 2,4,6‐trihydroxybenzophenone (246THB) by benzophenone synthase (BPS), a type III polyketide synthase (Liu et al., 2003). Subsequently, 3′‐hydroxylation and regioselective oxidative phenol couplings catalyzed by homologous cytochrome P450s (CYPs) result in the formation of either 1,3,5‐trihydroxyxanthone (135THX) or 1,3,7‐trihydroxyxanthone (137THX), the common precursors of plant xanthones (El‐Awaad et al., 2016). Further modifications via hydroxylation, methylation, glycosylation and prenylation lead to the formation of a plethora of xanthones (El‐Seedi et al., 2009; Remali et al., 2022; Schmidt et al., 2000; Wang et al., 2022). Our group has identified several aPTs that catalyze regiospecific forward prenylations of various xanthones (Fiesel et al., 2015; Nagia et al., 2019; Sayed et al., 2023). Variants of xanthone 4‐PT (*Hp*PT4px v1–v4) from *H. perforatum* attach a forward prenyl group at the C‐4 position of tri‐ and tetrahydroxyxanthones (Sayed et al., 2023). *Hs*PT8px and *Hs*PTpat from *H. sampsonii* catalyze two successive C‐8 prenylations of 1,3,6,7‐tetrahydroxyxanthone (1367THX) to yield the geminal diprenylated patulone (Nagia et al., 2019). Patulone is the immediate precursor of hyperixanthone A which arises from the attachment of a reverse prenyl group at the C‐4 position (Figure 1). The reverse aPT catalyzing this last biosynthetic step remained unidentified.

In this work, we confirm the occurrence of hyperixanthone A and its precursors in *H. sampsonii* and additionally detect their presence in *H. perforatum* roots. We report transcriptomics‐guided isolation of two orthologous candidate sequences encoding putative reverse aPTs. Upon heterologous expression and functional characterization, both enzymes show identical activities and strict substrate specificity towards patulone. In addition to the anticipated reverse prenylation at C‐4, the identified aPTs also catalyze forward prenylation at C‐2 of the xanthone skeleton. We rationalize the observed activities by modeling, docking, and site‐directed mutagenesis. The identified enzymes are localized to the plastid envelope and are phylogenetically related to xanthone‐specific aPTs. Finally, we achieve *in vivo* production of hyperixanthone A in *Saccharomyces cerevisiae* cells and *Nicotiana benthamiana* leaves.

## RESULTS

### Root extracts of *H. sampsonii* and *H. perforatum* contain hyperixanthone A

**Figure 2:**
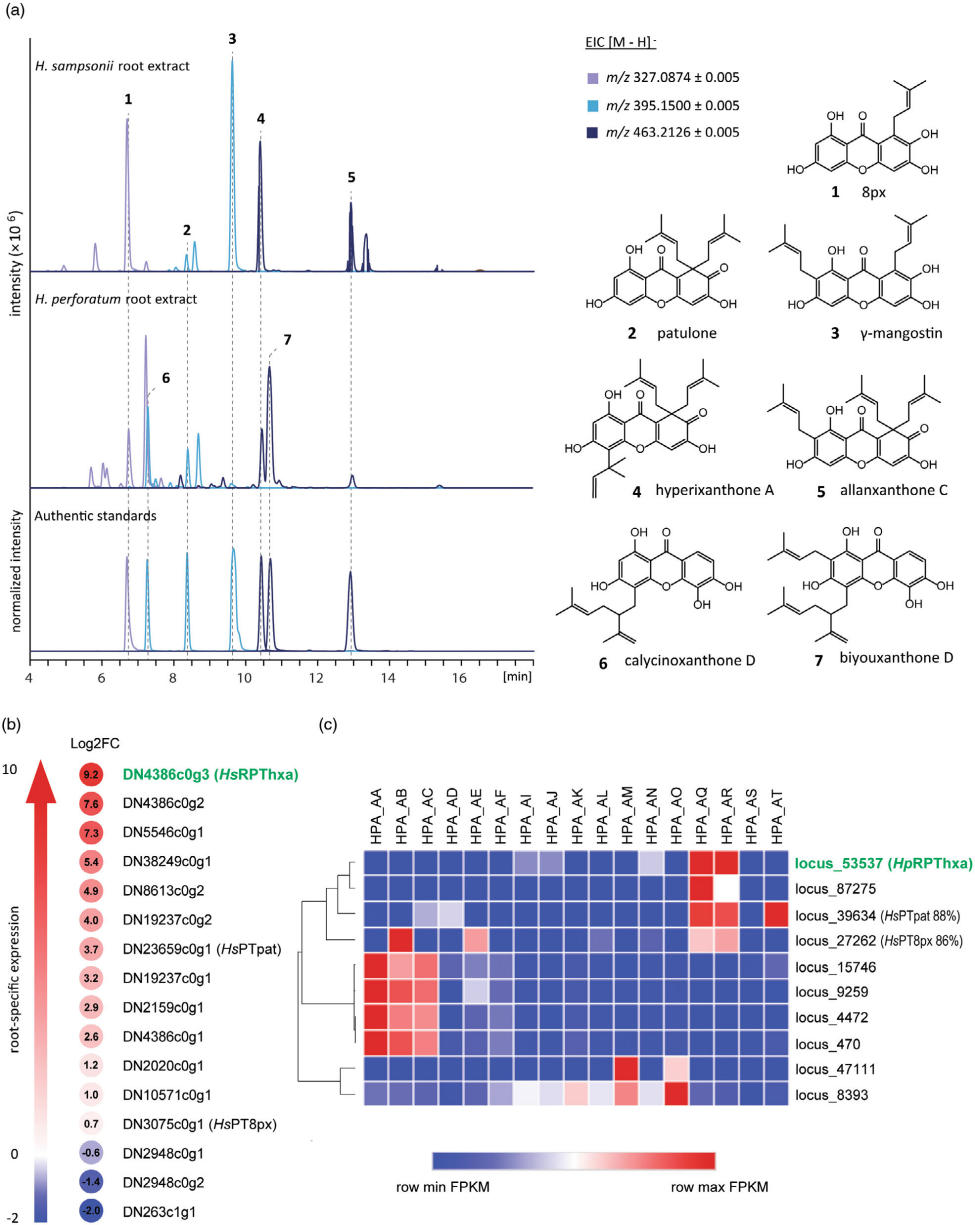
Mass‐guided metabolic profiling and transcriptomic analysis to select candidate *aPT* genes encoding reverse prenyltransferases. (a) Methanolic extracts from *H. sampsonii* and *H. perforatum* roots were analyzed by LC‐ESI‐HRMS. The extracted ion signatures correlating with mono‐, di‐, and triprenylated tetrahydroxyxanthones were annotated by either NMR analysis or comparison with authentic standards. Both preparations contained hyperixanthone A (4, *R*_t_ 10.39), allanxanthone C (5, *R*_t_ 12.92), and their direct biosynthetic precursors, 8PX (1, *R*_t_ 6.70) and patulone (2, *R*_t_ 8.34). While the extract from *H. sampsonii* also contained high amounts of γ‐mangostin (3, R_t_ 9.62), the two lavandulyl‐bearing compounds calycinoxanthone D (6, *R*_t_ 7.28) and biyouxanthone D (7, R_t_ 10.59) were exclusively identified in *H. perforatum*. (b) Calculated log2 fold changes in the expression levels of candidate reverse aPT‐coding sequences from shoot and root samples of *H. sampsonii*. Transcript DN4386c0g3 (shown in green) was selected for both its highly specific occurrence in roots and its high overall abundance compared with other root‐specific candidates. (c) Heatmap of RNA‐seq data from the Medicinal Plant Genomics Resource database (MPGR) showing differential expression of *H. perforatum* candidate aPT transcripts in different organs. Hierarchical clustering identified transcript hpa_locus_53537 (shown in green), which is upregulated in roots and co‐expressed with *Hs*PT8px and *Hs*PTpat homologs. HPA_AA, pistils, stamens, and sepals of mature flowers; HPA_AB, whole flower buds; HPA_AC, fully expanded mature flowers; HPA_AD, petals of fully expanded mature flowers; HPA_AE, pistils, stamens, and sepals of flower buds; HPA_AF, petals of flower buds; HPA_AI, whole mid‐aged leaves; HPA_AJ, whole old leaves; HPA_AK, whole young leaves; HPA_AL, portion of young leaves with light and dark glands; HPA_AM, portion of old leaves with only light glands; HPA_AN, portion of young leaves with only light glands; HPA_AO, portion of old leaves with light and dark glands; HPA_AQ, middle‐aged part of the roots; HPA_AR, oldest part of the roots; HPA_AS, dark glands of flower petals; HPA_AT, flower petals; FPKM, fragments per kilobase of transcript per million mapped reads.

To confirm the presence of hyperixanthone A in roots in the context of our endeavor to explore the biosynthetic background of plant reverse prenylation reactions, we performed metabolic profiling of the methanolic extract obtained from *H. sampsonii* roots. Liquid chromatography coupled to electrospray ionization high‐resolution mass spectrometry (LC‐ESI‐HRMS) focused on the annotation of xanthone‐related signals by evaluating their extracted ion signatures. We extracted ion peaks relevant to hyperixanthone A and its biosynthetic precursors 1,3,6,7‐tetrahydroxy‐8‐prenylxanthone (8PX) and patulone (Figure 1). Extracted ion chromatograms with [M–H]^−^ matching tetrahydroxyxanthone derivatives carrying C_5_ (*m/z* 327.0874), C_10_ (*m/z* 395.1500), or C_15_ (*m/z* 463.2126) residues showed multiple peaks (Figure 2a). Comparison with authentic standards allowed the unambiguous identification of 8PX (1, *R*_t_ 6.70), patulone (2, *R*_t_ 8.34), and γ‐mangostin (3, *R*_t_ 9.62). The structures of hyperixanthone A (4, *R*_t_ 10.39) and allanxanthone C (5, *R*_t_ 12.92) were verified by NMR analysis of root isolates. The recorded chemical shifts for 4 and 5 were consistent with previously published datasets (Azebaze et al., 2006; Xiao et al., 2008), disclosing a 8,8‐diprenyl‐1,3,6,‐trihydroxyxanthen‐7,9‐dione skeleton similar to 2 with either an additional reverse prenyl group attached at C‐4 in 4 or a forward prenyl group attached at C‐2 in 5 (Figures S3–S11). Thus, the accumulation of hyperixanthone A and its precursors, among other prenylated xanthones, in *H. sampsonii* roots was confirmed.

Likewise, a comparative analysis of the metabolite profile of *H. perforatum* roots was performed (Figure 2a). Although 4 had previously not been isolated from this plant, reports of reverse‐prenylated molecules can be found in the literature (Tusevski et al., 2017). Furthermore, several unknown 1,3,6,7‐tetrahydroxyxanthone derivatives have recently been detected in *H. perforatum* root samples (Sayed et al., 2023). In agreement with Sayed et al. (2023), our analysis revealed the two prominent lavandulyl‐bearing metabolites, calycinoxanthone D (6, *R*_t_ 7.28) and biyouxanthone D (7, *R*_t_ 10.59). Furthermore, both root extracts shared most of the annotated signals (1–5). Remarkably, our target compound 4 was one of the abundant extract constituents, proving its presence in *H. perforatum* for the first time.

In addition, extracted ion chromatograms with [M–H]^−^ matching trihydroxyxanthone derivatives with appended C_5_ (*m/z* 311.0925), C_10_ (*m/z* 379.1551), or C_15_ (*m/z* 447.2177) residues showed multiple minor peaks (Figure S12). However, the exact prenylation patterns of the compounds could not be identified.

### Transcriptomic analysis identifies candidate sequences encoding reverse prenyltransferases

*De novo* assembled transcriptomic data for *H. sampsonii* roots and shoots (Ernst et al., 2024) were mined for candidate aPTs potentially catalyzing the final reverse prenylation of 2 to yield 4. The amino acid sequences of the upstream enzymes, *Hs*PT8px and *Hs*PTpat, responsible for catalyzing the sequential *gem*‐diprenylation of 1,3,6,7‐tetrahydroxyxanthone (Nagia et al., 2019), were used as probes to identify homologs via a translated nucleotide BLAST search (tBLASTn). Since *HsPTpat* showed the highest expression in roots and xanthone metabolites exclusively accumulated in roots, only sequences with root‐specific expression profiles were considered as potential candidates. According to the calculated log2 fold changes (log2FC) in transcript abundance between root and shoot samples, transcript DN4386c0g3 showed the most specific occurrence in roots out of all identified sequences (Figure 2b, Table S1), and it included a full‐length ORF of 1176 bp. Its overall abundance was also the highest among the top three log2FC scores (Table S1). A homology search for each of the *H. sampsonii* transcripts against the assembled *H*. *perforatum* transcriptome publicly available in the Medicinal Plant Genomics Resource database (Góngora‐Castillo et al., 2012) yielded 10 homologous transcripts (Table S1). Hierarchical clustering of the expression data of those sequences in different organs (Figure 2c) revealed a transcript, hpa_locus_53537, whose translation product shared 86.3% amino acid sequence identity with the translation product of DN4386c0g3 from *H. sampsonii*. Interestingly, hpa_locus_53537 clustered together with *H. perforatum* transcripts hpa_locus_27262 and hpa_locus_39634, whose translation products shared 86.34 and 87.78% identity with *Hs*PT8px and *Hs*PTpat, respectively. The 5′‐end of hpa_locus_53537 lacked 825 nucleotides to the start codon. The sequence was extended through a blast analysis (BLASTn) against the publicly available assembled *H. perforatum* transcriptome of the 1000 Plant Transcriptomes Initiative (oneKP) (Carpenter et al., 2019). The transcript BNDE_scaffold_2094897 shared 96.9% identity with hpa_locus_53537 and had an ORF of 1125 bp. The BNDE_scaffold_2094897 missed a stretch of 48 bp towards the 5′ end, which was completed by the sequence hpa_locus_49015 from the MPGR database using the algorithm tBLASTn to give a full‐length ORF of 1173 bp. Both candidates, DN4386c0g3 and the full‐length version of hpa_locus_53537, were successfully amplified from root cDNA pools and tested in an aPT assay.

### Functional characterization reveals the first UbiA‐type reverse aromatic prenyltransferase

**Figure 3:**
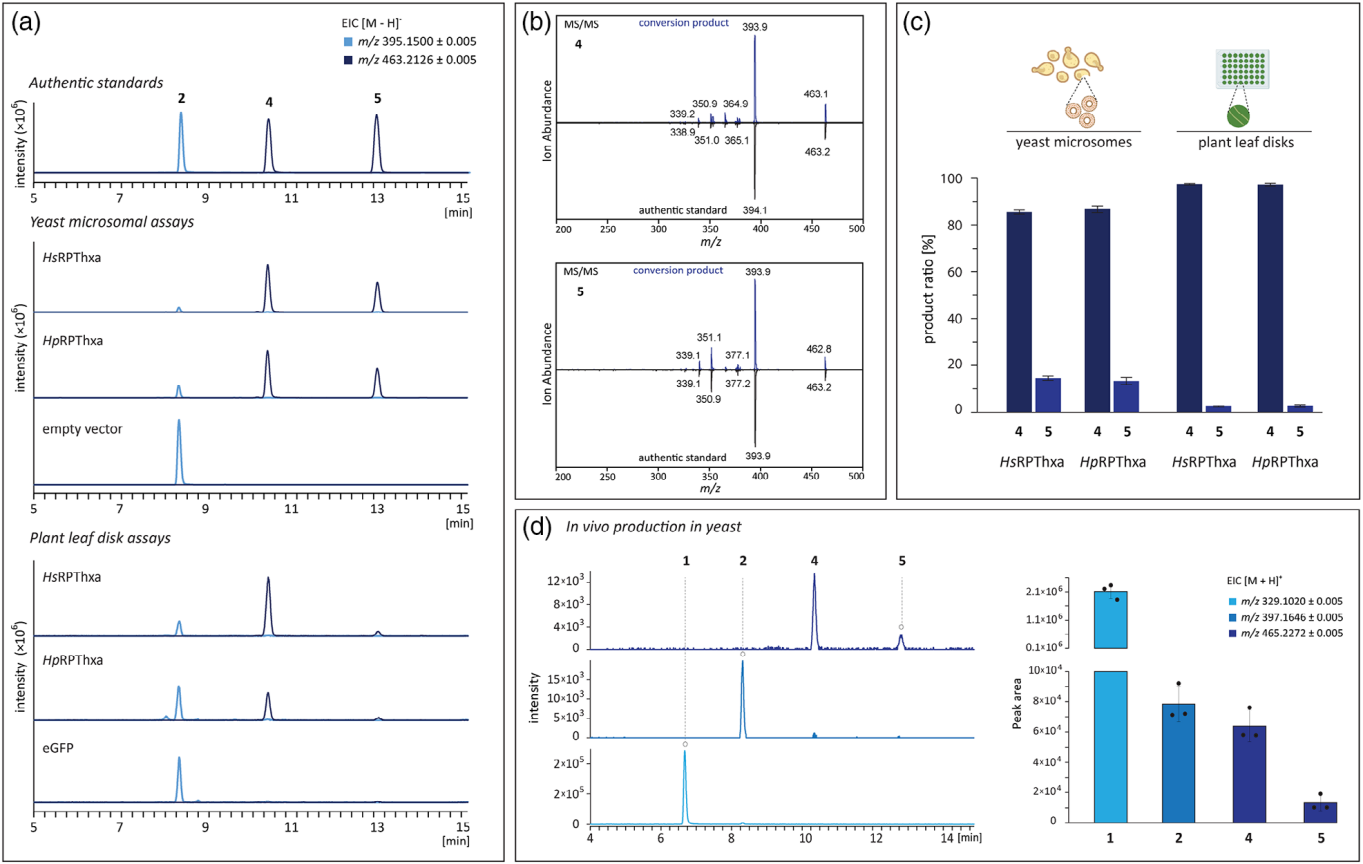
Functional characterization of *Hs*RPThxa and *Hp*RPThxa heterologously produced in *S. cerevisiae* cells and *N. benthamiana* leaf disks. (a) Extracted ion chromatograms of heterologous expression studies. Enzyme assays with microsomes containing *Hs*RPThxa or *Hp*RPThxa in the presence of acceptor 2 (*R*_t_ 8.34) and donor DMAPP yielded the primary product 4 (*R*_t_ 10.39) and the secondary product 5 (*R*_t_ 12.92). Meanwhile, control assays with microsomes from empty vector‐transformed cells showed no activity. Plant tissue assays comprising disks of transgenic *N. benthamiana* leaves and acceptor substrate 2 gave the same two conversion products 4 and 5, with 4 clearly dominating over 5. No conversion was observed in plants infiltrated with the eGFP control construct. (b) MS/MS fragmentation patterns of enzymatic products and structure‐elucidated standards. (c) Comparison of product formations in plant leaf disks and yeast microsome incubations. Data represent means ± SD obtained from three biological repeats. (d) Production of hyperixanthone A in *S. cerevisiae*, as illustrated by LC–MS extracted ion chromatograms and peak areas of products in cells co‐expressing the sequences *Hs*PT8px, *Hs*PTpat, and *Hs*RPThxa after feeding 1,3,6,7‐tetrahydroxyxanthone (48 h). Data represent means ± SD obtained from three biological replicates.

The candidate sequences obtained from *H. sampsonii* (DN4386c0g3) and *H. perforatum* (hpa_locus_53537) were individually cloned into the pESC‐URA vector, which allows the expression of target genes in *S. cerevisiae* under the control of the inducible GAL promoter. The microsomal fractions containing the heterologous translation products of the transcripts were isolated and used for enzyme assays containing compound 2 as acceptor and dimethylallyl pyrophosphate (DMAPP) as donor. Both aPTs accepted 2 as substrate, leading to the formation of two distinct products (Figure 3a). Both products had one additional prenyl group; however, the modes and locations of the prenyl attachment were different. The major reaction product was consistent with the C‐4 reverse‐prenylated compound 4, whereas the minor product 5 was due to a forward prenylation at C‐2. Neither enzyme accepted geranyl pyrophosphate (GPP) as a donor nor any of the potential acceptor substrates shown in Figure S13. Control reactions with microsomal fractions from empty vector‐transformed yeast cells lacked activity. The identities of the products were validated by comparing their MS/MS spectra to those of the structurally elucidated standards (Figure 3b). In accordance with their major conversion product, the proteins encoded by DN4386c0g3 and hpa_locus_53537 were named *Hs*RPThxa and *Hp*RPThxa, respectively.

Subsequent characterization of the full‐length enzymes established similar requirements in their physicochemical environment. The most efficient conversions were achieved in assays incubated at pH 9.0–9.5, employing magnesium ions as a cofactor (Figures S14 and S15). The optimal temperature varied slightly from 35°C for *Hs*RPThxa to 40°C for *Hp*RPThxa. Kinetic parameter studies on the basis of Michaelis–Menten saturation indicated comparable affinities of the enzymes towards acceptor substrate 2 for the formation of product 4 (Figure S16). *Hs*RPThxa and *Hp*RPThxa showed apparent K_M_ values of 52.40 ± 4.16 and 45.97 ± 4.43 μm, respectively. Both enzymes showed a comparatively lower affinity for the prenyl donor, DMAPP. The determined apparent K_M_ values were 102.21 ± 6.35 μm and 259.99 ± 22.88 μm for *Hs*RPThxa and *Hp*RPThxa, respectively. In contrast, the enzymes differed remarkably in their acceptor substrate affinities for the formation of product 5 (Table S2). As the N‐terminal region of UbiA PTs is known to affect enzyme activity (Munakata et al., 2019; Shen et al., 2012), we additionally studied a truncated version of *Hs*RPThxa lacking the initial 24 amino acids (*Hs*RPThxa‐tr). *Hs*RPThxa‐tr showed maximum activity at 40°C and pH 9.0–10.0. Compared to the full‐length version, *Hs*RPThxa‐tr exhibited higher affinity towards both the donor and the acceptor substrates (Figure S16, Table S2).

**Figure 4:**
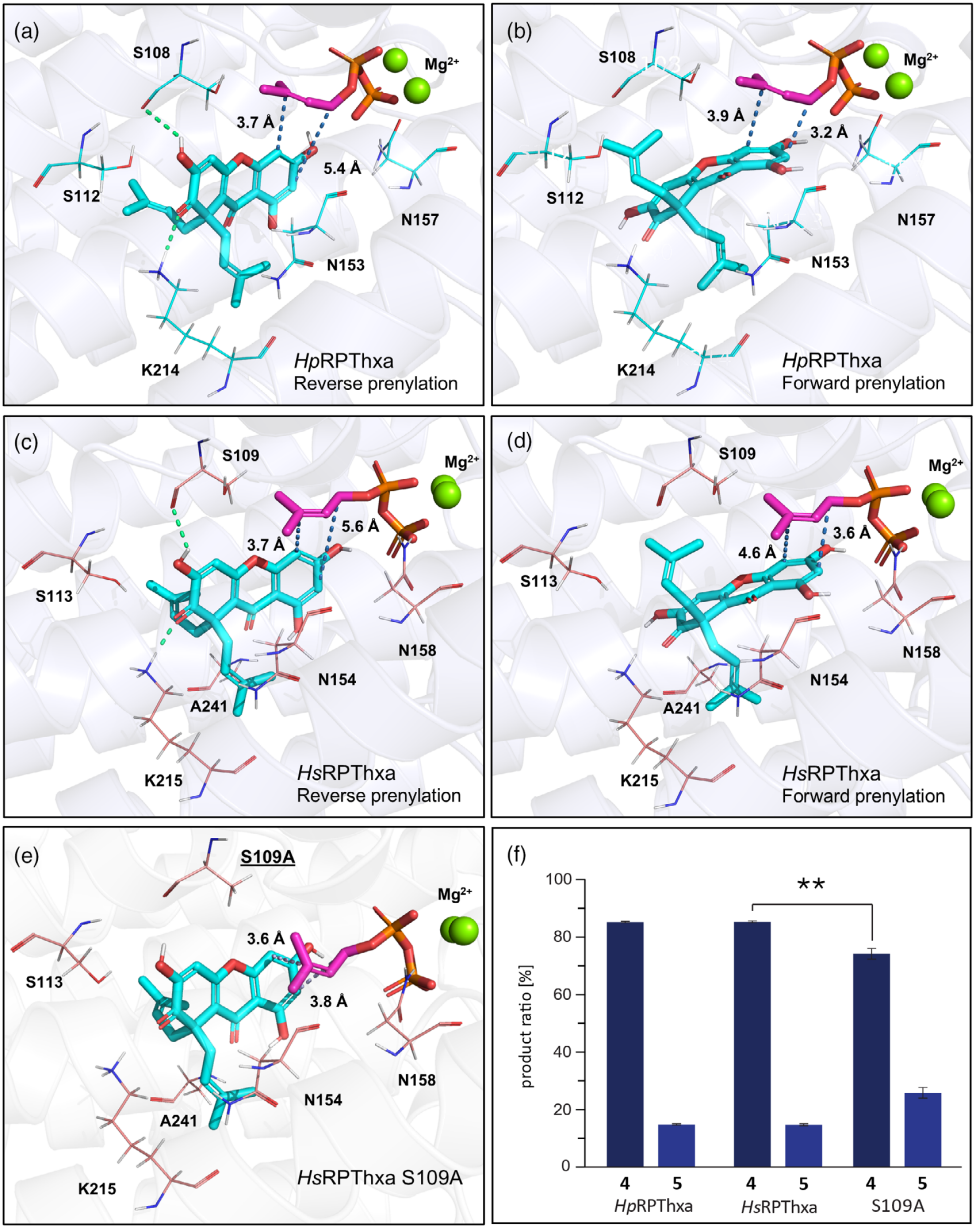
Catalytically competent substrate docking poses favoring either reverse or forward prenylation in the active site cavities of *Hp*RPThxa, *Hs*RPThxa, and the S109A variant of *Hs*RPThxa. (a) The catalytic docking pose of the donor‐acceptor complex in the active site cavity of *Hp*RPThxa, which is stabilized by hydrogen bonds with lysine 214 and serine 108, reveals that the catalytic distance favors reverse prenylation at C‐4. (b) The catalytic docking pose in the *Hp*RPThxa active site, which lacks the stabilizing effect of hydrogen bonds, shows the near‐attack distance for forward prenylation at C‐2. (c) The catalytic docking pose of the donor‐acceptor complex in the active site cavity of *Hs*RPThxa, which is stabilized by hydrogen bonds with lysine 215 and serine 109, reveals that the catalytic distance favors reverse prenylation at C‐4. (d) The catalytic docking pose in the *Hs*RPThxa active site, which lacks the stabilizing effect of hydrogen bonds, shows the near‐attack distance for forward prenylation at C‐2. (e) The catalytic docking pose of the S109A variant of *Hs*RPThxa, which exhibited an altered product ratio. (f) Comparison of product formations upon incubation of the yeast microsomes containing *Hp*RPThxa, *Hs*RPThxa, or *Hs*RPThxa S109A under saturation conditions of substrate 2 and DMAPP. Data represent means ± SD obtained from three biological repeats, **P‐value <0.01.

The ratios of products 4 to 5 were determined for each assay employing variable acceptor or donor concentrations (Figure S16). Under conditions used for kinetic parameter measurement, increasing the concentration of acceptor 2 slightly increased the percentage of the major product 4 for the full‐length and the truncated *Hs*RPThxa, but led to a decrease in the percentage of 4 in the case of *Hp*RPThxa (Figure S16a,c,e). A decrease in the formation of 4 was found for all variants (*Hs*RPThxa, *Hs*RPThxa‐tr and *Hp*RPThxa) with increasing concentrations of the prenyl donor (Figure S16b,d,f). Under standard assay conditions employing saturation concentrations of both donor and acceptor substrates, the product ratio of *Hs*RPThxa‐tr closely resembled those of full‐length *Hs*RPThxa and *Hp*RPThxa (Figure 4f).

### Heterologous expression in *N. benthamiana* confirms the activities of *Hs*RPThxa and *Hp*RPThxa

To verify the role of the identified enzymes in a plant system, the transcripts for *Hs*RPThxa and *Hp*RPThxa were transiently expressed in *N. benthamiana* leaves by agroinfiltration. Sections of transgenic leaves were incubated with substrate 2 before their metabolite content was analyzed by LC‐ESI‐HRMS. Both enzymes were functional in leaf disks and catalyzed the same transformations as observed with yeast microsomes (Figure 3a). Although expression levels were not normalized between the *Hypericum* species, analysis of three biological replicates each revealed that *Hs*RPThxa exhibited, on average, a 5.4‐fold higher activity in *N. benthamiana* than *Hp*RPThxa. No product accumulated in leaves transformed with the eGFP‐encoding control construct. Notably, the favored enzymatic reaction *in planta* was the reverse prenylation at C‐4, highlighting compound 4 as the major conversion product. The ratio of the products 4 and 5 changed from approximately 85:15 in yeast microsomes to 96:4 in plant leaf disks (Figure 3c).

### Concerted expression of three consecutive aPTs enables hyperixanthone A formation in yeast

Elucidation of the complete prenylation pathway leading to 4 prompted us to test baker's yeast as a production platform for polyprenylated xanthones. The *S. cerevisiae* strain AE9G, engineered to enable the production of monoterpenes (Fischer et al., 2011), was used for the co‐expression of the three consecutive aPTs (*Hs*PT8px, *Hs*PTpat, *Hs*RTPhxa) involved in hyperixanthone A 4 biosynthesis. Initially, no trace of 4 could be detected in yeast cells fed with 1,3,6,7‐tetrahydroxyxanthone when wild‐type *Hs*RPThxa was used, despite the fact that the microsomal fraction of the co‐transformed yeast cells exhibited the expected *in vitro* catalytic activity (Figure S17). To tackle this issue, we substituted the full‐length version of *Hs*RPThxa with the truncated version *Hs*RPThxa‐tr. This led to the formation of measurable amounts of the final products 4 and 5, with intermediate 1 being the dominant product (Figure 3d). The intermediates 1 and 2 predominantly accumulated within the cells, that is, 94.56 and 84.67%, respectively, of their total amounts were extracted from the cell pellet. The final products 4 and 5 were not detectable in the medium. Interestingly, quantification of yeast cell lysates indicated that the ratio of products 4 and 5 was 94:6. This ratio differed considerably from that obtained in *in vitro* incubations with yeast microsomes (85:15) but resembled that observed with *N. benthamiana* leaf disks (96:4).

### Molecular modeling and docking studies rationalize the preferred formation of the reverse‐prenylated product

Mechanism‐based substrate docking simulations were conducted to rationalize the different regioselectivities and prenylation modes of the *Hp*RPThxa‐ and *Hs*RPThxa‐catalyzed reactions. Initially, we predicted and validated 3D models for both *Hp*RPThxa and *Hs*RPThxa using AlphaFold2 (Jumper et al., 2021). Subsequently, homology modeling was conducted using YASARA (Krieger et al., 2002) to simulate the binding of Mg^2+^ and DMAPP. In the substrate docking simulations, we assessed the catalytically competent binding poses of the substrate patulone 2 in the *Hp*RPThxa (Figure 4a,b) and *Hs*RPThxa (Figure 4c,d) active sites by applying geometric and catalytic criteria according to the established catalytic mechanism of aPTs (Luk & Tanner, 2009). The docking results predicted two distinct catalytically competent binding poses that fulfill the respective criteria for C‐2 forward and C‐4 reverse prenylation reactions (Figure 4). Calculated binding energies within *Hs*RPThxa were in favor of the reverse‐specific binding mode as the substrate conformation is highly stabilized by hydrogen bonds with a binding energy of −9.67 kcal mol^−1^ (Figure 4c), compared to the scenario of the forward reaction with a binding energy of −8.23 kcal mol^−1^ (Figure 4d). The calculated catalytic distance and angles criteria correlate with the experimentally observed preference of the reverse prenylation (Figure 3c). *In silico* site‐saturation mutagenesis was applied to the *Hs*RPThxa active site residues within a radius of 4.5 Å of the prenyl donor or acceptor substrate to investigate their possible contribution to stabilizing the alternative binding scenarios (Table S3). In total, 500 variants were assessed and ranked based on the improvement of their stability and substrate affinity (Data S1, Table S4). The top ranked poses were further evaluated for the improvement of the catalytically competent criteria for either forward or reverse prenylation. Among the *in silico* screened variants, S109A and N154W were predicted to destabilize the reverse‐specific binding mode, while N158Y was predicted to interfere with the forward prenylation (Figure 4e, Figure S18). The suggested single mutations were introduced to *Hs*RPThxa and the enzyme variants were tested for activity. While the N154W and N158Y variants showed a complete loss of enzymatic activity, S109A significantly changed the ratio of the products 4 and 5 from 85:15 to 74:26 in favor of the forward prenylation product 5, as predicted by the model (Figure 4f).

### Phylogenetic analysis reveals the relationship between the RPThxa enzymes and xanthone‐specific aPTs

**Figure 5:**
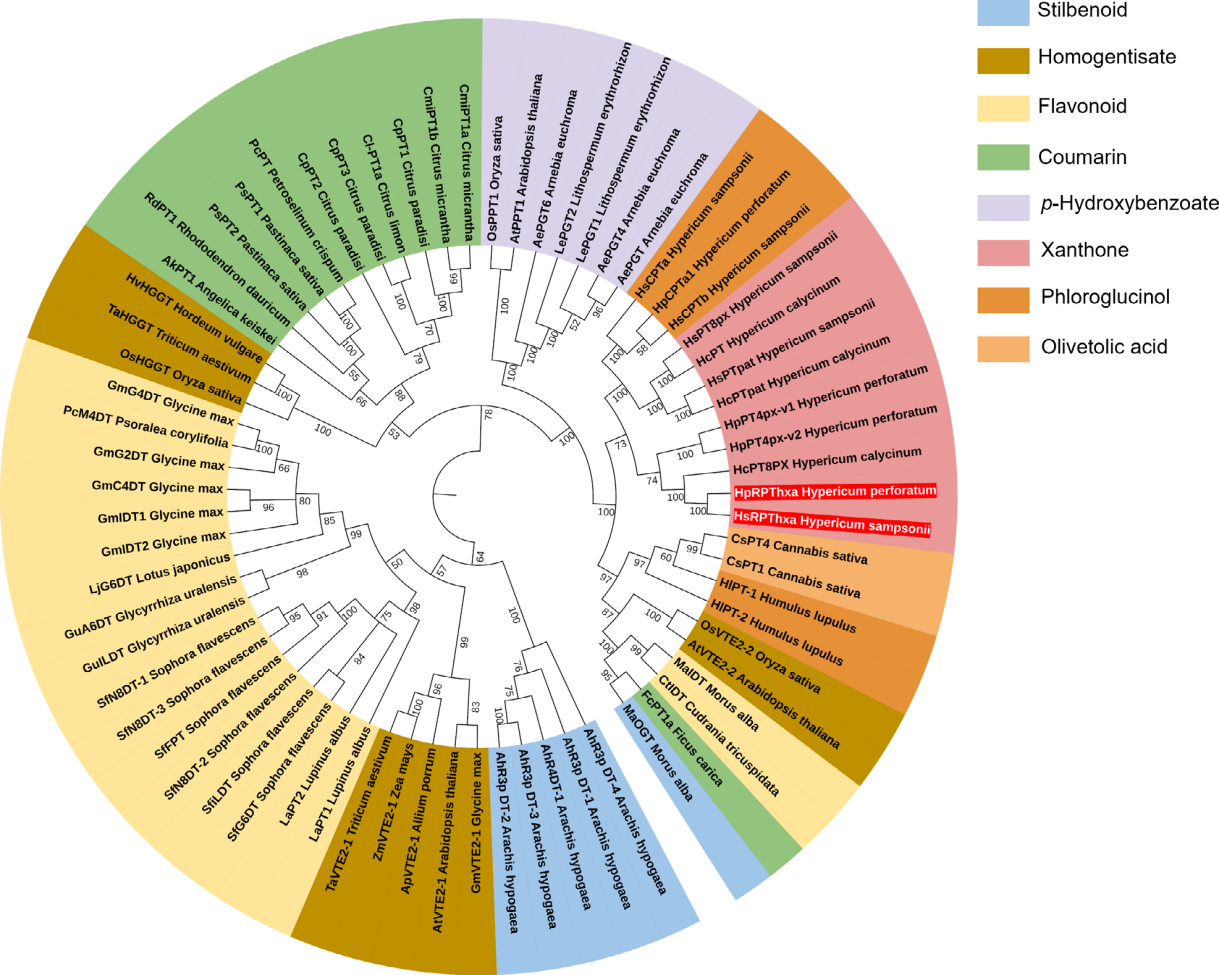
Phylogenetic tree depicting the evolutionary relationships between aPTs from a diversity of plant species. *Hs*RPThxa and *Hp*RPThxa (this work) are highlighted in red. Clades are color‐coded according to their preferred prenyl acceptors. The maximum likelihood tree was constructed using amino acid sequences of previously identified aPTs employing the MEGA 7 software with the support of 1000 bootstraps and a JTT‐f model. The bootstrap values are indicated at the branch points.

A phylogenetic tree was designed as previously described in Sayed et al. (2023). The accession numbers of the sequences included are listed in Table S5. In the maximum likelihood tree (Figure 5), the *Hs*RPThxa and *Hp*RPThxa sequences clustered together with previously identified xanthone‐specific aPTs of *Hypericum* species, including those responsible for the upstream prenylation steps in hyperixanthone A biosynthesis (Fiesel et al., 2015; Nagia et al., 2019; Sayed et al., 2023).

### C‐terminal reporter fusion of *Hs*RPThxa is localized to the chloroplast envelope

**Figure 6:**
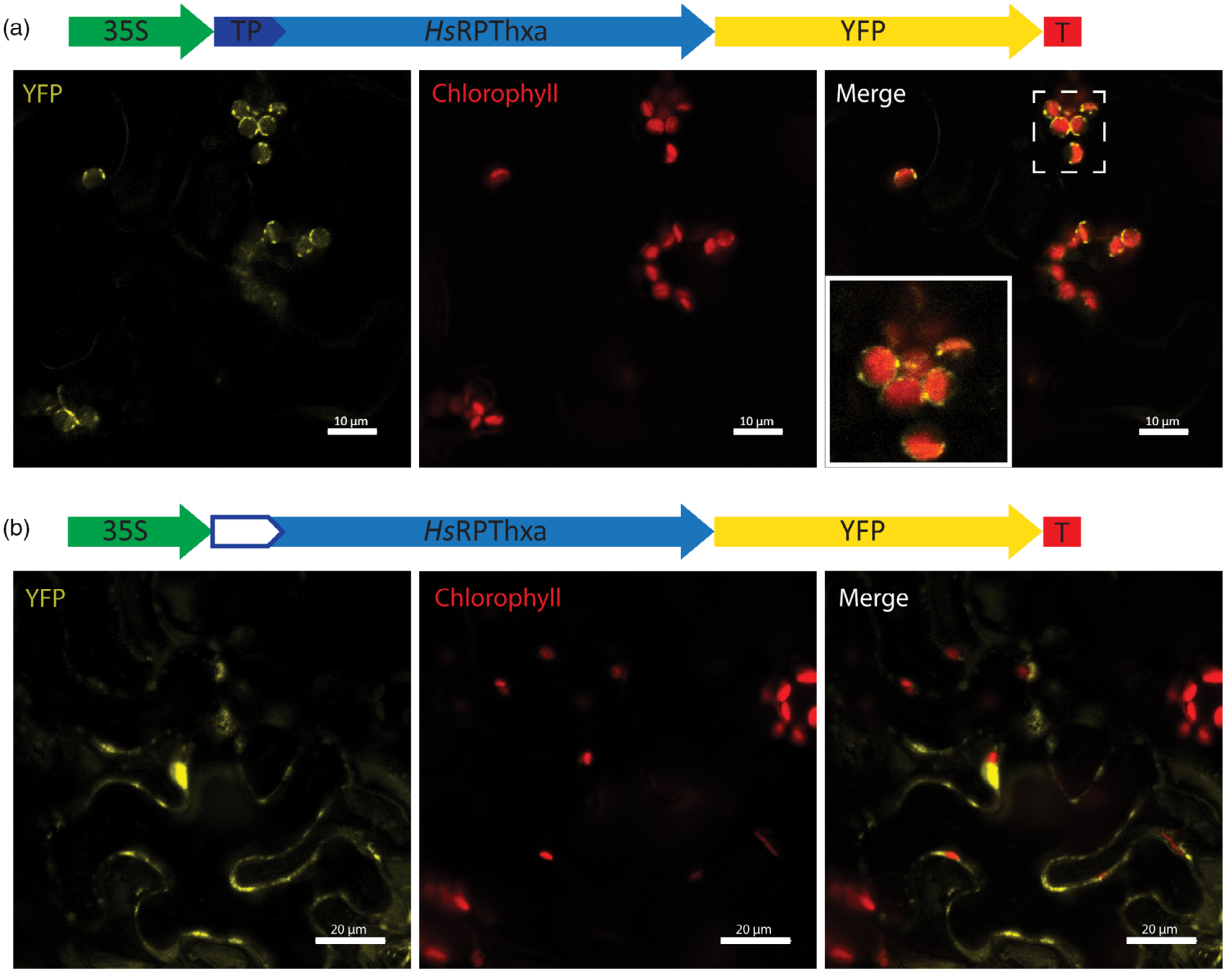
Subcellular localization of *Hs*RPThxa in epidermal cells of *N. benthamiana*. Translational fusions of *Hs*RPThxa to the C‐terminus of a YFP reporter were examined by confocal laser scanning microscopy. (a) The fluorescence signature of the fusion protein with an intact transit peptide matched the outline of chlorophyll autofluorescence, indicating targeting of *Hs*RPThxa‐YFP to the chloroplast envelope. (b) Truncation of the N‐terminal transit peptide disrupted the functional localization and led to accumulation of the fusion protein in the cytoplasm and nucleus. 35S, constitutive promoter from Cauliflower Mosaic Virus; TP, transit peptide; YFP, yellow fluorescent protein; T, terminator sequence.

The spatial distribution of *Hs*RPThxa in cellular compartments of *N. benthamiana* leaves was investigated by confocal laser scanning microscopy. Epidermal cells transiently produced a reporter (yellow fluorescent protein, YFP) fused to the C‐terminus of *Hs*RPThxa. The emitted YFP fluorescence matched the outline of the plastidial autofluorescence (Figure 6a). The functional targeting of *Hs*RPThxa‐YFP to the chloroplast envelope depended on the presence of an N‐terminal transit peptide. Truncation of the putative leader sequence disrupted the localization (Figure 6b).

## DISCUSSION

Prenyltransferases (PTs) contribute to the biosynthesis of a diversity of primary and specialized metabolites, including phenolic compounds, in bacteria, fungi, and plants. Phenolic compounds are primed by prenylation for subsequent modifications, such as oxidation, cyclization, and dimerization, which ultimately contribute to expanding the metabolic profile of host organisms and present promising avenues for the modification of lead compounds (Alhassan et al., 2014; Wang et al., 2024; Yazaki et al., 2009). While soluble microbial aPTs are known to catalyze both forward and reverse *C*‐, *N*‐, and *O*‐prenylation reactions, almost all known plant aPTs (UbiA‐type) catalyze forward prenylations, predominantly on C‐ and rarely on O‐atoms (de Bruijn et al., 2020; Munakata et al., 2021). Recently, Zheng et al. (2024) identified the first plant PT that catalyzes a reverse geranylation reaction of a nonaromatic carbon followed by decarboxylation of *para*‐coumaric acid. Despite the prevalence of plant reverse‐prenylated phenolics (Figure S2a), no UbiA‐type aPT catalyzing reverse prenylation of aromatic carbons has been identified to date. Herein we demonstrate the identification and functional characterization of two plant aPTs from *H. sampsonii* (*Hs*RPThxa) and *H. perforatum* (*Hp*RPThxa), which catalyze a reverse prenylation reaction in xanthone metabolism.

In the present study, metabolic profiling unequivocally detected both 4 and 5, along with their precursors, in *H. sampsonii* and *H. perforatum*. Furthermore, the examined root extracts contained additional polyprenylated xanthone derivatives, showcasing *Hypericum* species as valuable sources for the isolation of aPTs that catalyze yet unexplored prenylation reactions (Figure S19). The focus of metabolic profiling was primarily directed toward tetrahydroxyxanthone derivatives, which include 4. Polyprenylated xanthones with a tetrahydroxyxanthone core represent the most predominant subgroup of reverse‐prenylated metabolites in *Hypericum* (Figure S20).

The predominant accumulation of polyprenylated xanthones in the roots of *H. perforatum* and *H. sampsonii*, as opposed to their absence from the shoots, guided our bioinformatic efforts to identify genes encoding candidate reverse aPTs. We targeted transcripts overexpressed in the roots, which encode homologous aPTs in both species. Previously, the well‐established tendency of polyprenylated xanthones to accumulate in the roots of *H. perforatum* led to the isolation of *Hp*PT4px, a xanthone‐specific aPT. Interestingly, the gene expression pattern of *Hp*RPThxa accepting a diprenylated xanthone substrate slightly differed from that of *Hp*PT4px acting on non‐prenylated xanthone substrates. Specifically, *HpPT4px* exhibited a preference for expression in the leaves, followed by the roots (Sayed et al., 2023). In contrast, *HpRPThxa* transcripts were primarily present in the roots, as inferred by RNA‐seq data. Conversely, aPT genes responsible for the biosynthesis of type‐A and type‐B polycyclic polyprenylated acylphloroglucinols (PPAPs) were highly expressed in the shoots of *H. sampsonii* (Ernst et al., 2024). A simultaneous comparison between homologous sequences and their expression patterns in both species helped us narrow down the number of potential candidates, leading to the identification of the target RPTs with minimal screening effort. This approach can be applied to identify further xanthone‐ and phloroglucinol‐specific aPTs (Figure S19).

The products of *Hs*RPThxa were consistent among the expression systems employed, namely *in vitro* yeast microsomes and *in vivo N. benthamiana* leaf disks and yeast resting cells. However, the ratio between the major reverse and the minor forward prenylated products varied. The *in vivo* systems produced a higher reverse‐prenylated product ratio compared to yeast microsomes. The product profile in transgenic leaf disks demonstrated that C‐4 reverse prenylation is the principal function of *Hs*RPThxa. The *in vivo–in vitro* discrepancy in yeast expression might be related to the truncation of the N‐terminal transit peptide, along with the relatively low‐donor concentration *in vivo*, which favors formation of 4 (Figure S16). Interaction with co‐expressed upstream aPTs (*Hs*PT8px and *Hs*PTpat), which appear to physically interact within the membranes (Nagia et al., 2019), or divergent insertions of the expressed *Hs*RPThxa into chloroplast envelope and yeast intracellular membranes may also contribute to the observed difference in product ratios. A further explanation may be attributed to the effect of pH on product ratios, with the slightly basic reaction conditions used for *in vitro* assays favoring the formation of 5 (Figure S14c), compared with the neutral to slightly acidic medium predominating *in vivo* (Martinière et al., 2013). The occurrence of 5 among the major polyprenylated xanthones in *H. sampsonii* may originate from the inadvertent side reaction of *Hs*RPThxa. Nevertheless, we cannot dismiss the potential involvement of a yet unidentified aPT in catalyzing the final step of the biosynthesis of 5.

Having all involved aPTs in the biosynthesis of 4 identified and characterized, we explored the opportunity to biotechnologically produce 4 in yeast cells via three consecutive prenylation reactions upon feeding 1367THX. No product was detected when yeast cells were transformed with the wild‐type aPTs, probably due to their mislocalization compared to the compartmentalization in their native host. Only after removal of the transit peptides was the final product detectable, exclusively inside the cells. The accumulation of prenylated compounds within cells versus their excretion into the medium depends on both the compound class and the host cell type. For example, prenylated flavonoids accumulate within mammalian cells potentially due to increased hydrophobicity, improved membrane affinity, and interaction with membrane proteins (Terao & Mukai, 2014). In contrast, a large portion of the monoprenylated phenylpropanoid drupanin is excreted from the producing transgenic *Saccharomyces cerevisiae* cells (Munakata et al., 2019). In our work, prenylated xanthones accumulated preferentially (more than 80%) inside the producing yeast cells, suggesting the need for an active transport mechanism for their efflux. Despite the low yield, this experiment presents a proof‐of‐concept for the possible production of polyprenylated xanthones in yeast. *In vivo* prenylation in yeast cells demands careful adjustment of metabolic flux to effectively replenish the prenyl donor, presenting a complex challenge. Previous trials for the *de novo* production of the monoprenylated chalcone xanthohumol in yeast identified the prenylation reaction as the key limiting step in the pathway (Yang et al., 2024). Enhancements via removal of variable lengths of the N‐terminal transit peptide, codon optimization, fine‐tuning of enzyme activities by protein engineering, and variation of cultivation conditions are expected to improve the yield (Munakata et al., 2019; Yang et al., 2024). Moreover, alternative hosts such as *Komagataella phaffii* and *Yarrowia lipolytica* have recently been explored to overcome the limitations associated with heterologous production of prenylated metabolites in *S. cerevisiae* (Bamba et al., 2024; Liu, Zhu, et al., 2025). In the triple prenylation cascade under consideration, our observations indicate that the geminal diprenylation is most probably the limiting step, with the reverse prenylation promptly following the formation of 2, as indicated by the quantification of conversion intermediates (Figure 3d).

Both soluble and membrane‐bound UbiA‐type PTs operate via a Friedel–Crafts alkylation‐like mechanism. Cleavage of the pyrophosphate group of the prenyl donor results in the formation of an intermediate carbocation, which is subsequently attacked by the nucleophilic aromatic prenyl acceptor in an electrophilic aromatic substitution reaction (Cheng & Li, 2014; Luk & Tanner, 2009). Mechanistic studies on the basis of the co‐crystallized ternary prenyl donor‐acceptor complex of the DMATS‐type aPT FgaPT2 revealed that [3,3]‐sigmatropic rearrangements may play a role in the catalysis of reactions with different regioselectivities and prenylation modes. In the case of FgaPT2, this would explain why, despite the equal distance of the prenyl donor's C‐1 and C‐3 to the indole moiety of the acceptor substrate, the enzyme catalyzed only the forward prenylation of the poorly nucleophilic C‐4 position. In contrast to the C‐3 and C‐4 positions of the indole ring in the FgaPT2 example, the two carbon atoms targeted in the *Hp*/*Hs*PThxa‐catalyzed reactions are equally nucleophilic, which makes the catalytic hypothesis involving a [3,3]‐sigmatropic rearrangement less favorable (Luk et al., 2011; Metzger et al., 2009; Tanner, 2015). The alternative regioselectivities and prenylation modes observed for *Hp*/*Hs*RPThxa were rationalized by protein modeling and substrate docking, which revealed catalytically competent binding modes for both forward and reverse prenylation. In the reverse‐specific binding scenario, the magnesium‐mediated binding of the prenyl donor's pyrophosphate moiety places C‐3 of the prenyl group in close proximity to C‐4 of the acceptor substrate patulone 2. This mechanism is stabilized by hydrogen bonding and leads to the formation of hyperixanthone A 4. Notably, this binding scenario is energetically favored over an alternative pose, in which C‐1 of the prenyl group is positioned close to C‐2 of the patulone skeleton, which is not stabilized by hydrogen bonding and results in allanxanthone C 5 formation. A similar explanation was previously employed to justify alternative prenylation modes for the soluble prenyltransferase AmbP3, which catalyzes both forward and reverse prenylations at C‐2 of the hapalindole compounds A and G, respectively (Wong et al., 2018). The observed differences in the apparent K_M_ values for the formation of the products 4 and 5 (Table S2) further support the presence of distinct binding poses within the active site cavity, which appear to govern the biosynthesis of either the one or the other product. The *Hs*RPThxa model was used to guide site‐directed mutagenesis aiming at investigating the residues contributing to regioselectivity and acceptor binding mode of RPTs. Of the three mutations proposed by *in silico* site‐saturation mutagenesis, two changes resulted in enzyme inactivation. This suggests that the mutated residues likely play critical roles in facilitating substrate entry, product release, or interaction with magnesium ions, as observed with the N185Y variant (Figure S18a). However, the S109A variant was an active enzyme and exhibited an altered product ratio, further validating the accuracy of the model.

Recent studies have identified catalytic mechanisms and key residues that contribute to the regioselectivity of plant UbiA‐type aPTs. For example, substrate‐induced conformational changes were shown to modulate the regioselectivity of white mulberry (*Morus alba*) aPTs in the prenylation of moracin M. Rational mutations guided by structural models of multiple *Ma*PTs led to the introduction of a double mutation to *Ma*PT27, shifting its regioselectivity from C7‐ to C5‐prenylation, as catalyzed by *Ma*PT29 (Liu, Tao, et al., 2025). Ernst et al. (2024) demonstrated that inverted binding modes of the substrate kolanone within the active site cavities of *Hs*CPTa and *Hs*CPTb from *H. sampsonii* resulted in alternative prenylative cyclization reactions leading to the formation of type‐A and type‐B bicyclo[3.3.1]nonanes, respectively. Domain swapping followed by reciprocal site‐directed mutagenesis resulted in switching the regiospecificity of *Hs*CPTa to form type‐B products (Ernst et al., 2024). Han et al. (2025) employed a similar strategy to pinpoint mutations that altered the regioselectivity of Apiaceae aPTs involved in coumarin biosynthesis. Interestingly, the region extending from 55 AA upstream to 10 AA downstream of the conserved first aspartate‐rich motif (NxxxDxxxD) was most influential in the previous studies (Ernst et al., 2024; Han et al., 2025; Liu, Tao, et al., 2025). Likewise, the S109A mutation described in this work is located 49 AA upstream of the motif's boundary, highlighting the significance of this region in modulating the regioselectivity of plant UbiA‐type aPTs.

Phylogenetic analysis revealed that *Hp*/*Hs*RPThxa clustered together with other xanthone‐specific aPTs. This observation strongly suggests that reverse prenylation is a recent modification, which likely occurred after the specialization of aPTs for the conversion of xanthone substrates in the order Malpighiales. The recently unveiled *O*‐prenylation reaction catalyzed by plant aPTs was also found to have evolved in parallel in different plant lineages (Munakata et al., 2021). It would be intriguing to investigate the evolutionary pathway of reverse prenylation in other orders, such as Fabales and Rosales, which produce reverse‐prenylated chalcones and flavonoids (Figure S2a).

In conclusion, we identify a non‐canonical plant RPT and thereby augment the repertoire of reactions catalyzed by membrane‐bound UbiA‐type plant aPTs. We reveal the molecular basis for the biosynthesis of reverse‐prenylated xanthones in plants and expand the available biotechnological tools for their modification. In the future, aPTs are anticipated to be utilized for chemoenzymatic synthesis of new prenylated derivatives (An et al., 2023; Yu & Li, 2012). The present work constitutes a basis for identifying additional aPTs and exploring the regioselectivities and prenylation mode determinants. This will help further exploit their catalytic potential and design novel biocatalysts, opening new avenues for innovation in natural product modification and pharmaceutical discovery.

## METHODS

### Plant material

Pot plants of *Hypericum sampsonii* Hance (Hypericaceae) were grown as previously described (Ernst et al., 2024). For the isolation of prenylated xanthone metabolites, roots of 4‐week‐old plants were lyophilized prior to extraction. Roots of *Hypericum perforatum* were collected and used as previously stated (Sayed et al., 2023).

### Extraction and isolation of prenylated xanthones

Lyophilized *H. sampsonii* root material (15 g dry weight) was homogenized using a MM400 mixer mill (Retsch, Haan, Germany). The powder was evenly divided into twelve portions and extracted three times with 50 ml of methanol. After centrifugation at 9000 ***g*** for 10 min, the three portions were combined and passed through cellulose filter paper to remove residual debris. After evaporation under nitrogen gas, the crude extract (1.7 g) was fractionated by semi‐preparative HPLC using a Waters 2795 separation module equipped with a Waters 2996 photodiode array detector and a Waters Fraction Collector III (Waters, Milford, USA). HPLC conditions were as follows. A reversed‐phase EC NUCLEODUR column (HTec, 10 × 250 mm, 5 μm; Macherey‐Nagel, Düren, Germany) was used at a flow rate of 3.5 ml min^−1^ with the following binary gradient conditions: 0–2 min 85% B, 2–27 min 85–95% B (A, water; B, acetonitrile; both containing 0.1% formic acid). After brief LC–MS analysis, the fractions containing mass and UV signatures typical of polyprenylated 1367THX derivatives were subjected to a second round of purification, yielding hyperixanthone A (5.4 mg) and allanxanthone C (1.5 mg).

### NMR spectroscopy

NMR spectra were measured on a 400 MHz Bruker Advance III HD spectrometer (Bruker Biospin, Rheinstetten, Germany). The acquisition of ^1^H NMR, ^13^C NMR, ^1^H–^1^H COSY, ^1^H–^13^C HSQC, and ^1^H–^13^C HMBC data was accomplished using standard Bruker pulse programs. CDCl_3_ and C_3_D_6_O were used as solvents for the measurements of hyperixanthone A and allanxanthone C, respectively. NMR spectra were referenced to the residual solvent signals at δ_H_ 7.24 and δ_C_ 77.23 ppm for CDCl_3_ or δ_H_ 2.05 and δ_C_ 206.68 ppm for C_3_D_6_O. Spectrometer control, data acquisition, and processing were performed using the Bruker TopSpin 3.6.1 software.

### Commercial molecular biology services and products

All modified and unmodified primers were synthesized by Eurofins Genomics (Ebersberg, Germany). Sequencing of DNA samples was performed by Microsynth Seqlab (Göttingen, Germany). PCR reactions were carried out using either PCRBIO HiFi polymerase (PCR Biosystems, London, UK) or PfuTurbo Cx HotStart DNA polymerase (Agilent Technologies, Santa Clara, CA, USA) in order to read through uracil. PCR cleanup and isolation of DNA fragments from agarose gels was performed using the innuPREP DOUBLEpure kit (Analytik Jena, Jena, Germany). Restriction enzymes were purchased from Thermo Fisher. T4 DNA ligase (Thermo Fisher Scientific, Dreieich, Germany) was used to facilitate ligation reactions. Isolation of plasmid DNA was done with the QIAprepTM Spin Miniprep kit (Qiagen, Hilden, Germany). Unless indicated otherwise, all reactions using commercial products were performed in accordance with the guidelines provided by the manufacturer.

### RNA extraction and cDNA synthesis

*H. sampsonii* and *H. perforatum* root materials were freshly harvested, snap frozen, and homogenized in liquid nitrogen using a mortar and pestle. Powder aliquots (100 mg) were used for the extraction of total RNA employing the InviTrap Spin Plant RNA mini kit (Invitek Molecular, Berlin, Germany). After the removal of any residual genomic DNA impurities by digestion with DNase I (Thermo Fisher Scientific, Dreieich, Germany), the reaction mixture was purified using the Monarch RNA Cleanup kit (New England Biolabs, Ipswich, MA, USA). First‐strand cDNA synthesis was carried out with 1 μg total RNA using the RevertAid H Minus Reverse Transcriptase (Thermo Fisher Scientific, Dreieich, Germany) in the presence of both oligo(dT) and random hexamer primers. The cDNA obtained was used as a template to amplify candidate genes.

### Molecular cloning

For the assembly of yeast expression constructs, DNA fragments were amplified using overhang primers (Table S6), which contained restriction sites suitable for traditional restriction‐ligation cloning into MCS1 of pESC vectors (*Eco*RI/*Pac*I).

For the assembly of pCambia2300u plant expression constructs (Nour‐Eldin et al., 2006), a modified version of the ligation‐independent USER strategy (Bitinaite et al., 2007) was adopted as previously described (Ernst et al., 2024) using the uracil‐containing primers shown in Table S6.

### Functional expression in *Saccharomyces cerevisiae*

Sequence‐confirmed yeast expression constructs were transferred to *S. cerevisiae* INVSc1 cells (Thermo Fisher Scientific, Dreieich, Germany) and recombinant microsomal fractions were prepared as previously described (Ernst et al., 2024). Microsomal preparations were routinely stored at −80°C before use.

### Enzyme assay and characterization

Incubation and characterization of aPTs were carried out as described previously (Ernst et al., 2024).

### Transient gene expression in *N. benthamiana*

Sequence‐confirmed plant expression constructs were transferred to *Agrobacterium tumefaciens* EHA105 cells as described previously (Ernst et al., 2024).

### *N. benthamiana* leaf disk assays

To confirm the activities of the identified aPTs *in planta*, the previously described *N. benthamiana* leaf disk assay strategy was used (Ernst et al., 2024; Kamileen et al., 2022).

### *In vivo* hyperixanthone a production in yeast

*Saccharomyces cerevisiae* AE9G (Fischer et al., 2011), co‐transformed with the recombinant plasmids pESC‐URA‐*HsPT8px*‐*HsPTpat* and pESC‐HIS‐*HsRPThxa*, were used to inoculate 10 ml of histidine‐ and uracil‐deficient SGI medium (100 ml flask) to start the pre‐culture. After incubation for 24 h at 30°C and 200 rpm, the main cultures were started by inoculating 150 ml of YPGE medium (1 L flask) to an OD_600_ of 0.02. The main cultures were incubated for 24–30 h until glucose depletion. Afterwards, protein expression was induced by adding 2% of sterile‐filtered galactose, and the cultures were further incubated for 24 h before cell harvesting by centrifugation at 2375 ***g*** for 5 min. Production of hyperixanthone A was achieved using resting yeast cells. The cell pellets were resuspended in 15 ml of 1 M Tris–HCl pH 7 buffer, followed by separation into 3 × 5 ml cultures (100 ml flask). Subsequently, 0.2 mM 1367THX was added to each culture, and the cultures were incubated for 24 h at 37°C and 200 rpm. Afterwards, the cells were harvested by centrifugation at 2375 ***g*** for 5 min, washed twice with 5 ml 1 M Tris–HCl pH 7 buffer. The supernatant and pooled wash fractions were extracted with equal volumes of ethyl acetate, evaporated to dryness, resuspended, and analyzed by HPLC. The cell pellets were resuspended in 3 ml methanol and disrupted by vigorous shaking with an equal volume of glass beads (0.45 mm) for 20 min using the bead mill MM400 (Retsch, Haan, Germany). After centrifugation at 2375 ***g*** for 5 min, the supernatant was transferred into a new tube. These extraction steps were repeated, and the supernatant was pooled and evaporated to dryness under nitrogen. The residues were redissolved in methanol and analyzed by LC‐ESI‐HRMS.

### Analytical methods

HPLC‐DAD analysis was performed on either a VWR (Hitachi High‐Tech Corporation, Tokyo, Japan) LaChrom Elite machine (L‐2130 pump, L‐2200 autosampler, and L‐2455 diode array detector) or an Agilent 1260 Infinity system (G1311C quaternary pump, G1329B autosampler, and G4212A diode array detector). Operation of the two HPLC systems was accomplished using either EZChrom Elite 3.2.2 or ChemStation for LC B.04.03 (both Agilent). All samples were analyzed using a ZORBAX Eclipse Plus column (C18, 4.6 × 100 mm, 3.5 μm; Agilent). The following HPLC conditions were used. As a general analytical method (HPLC method 1), 0.1% formic acid in water (A) and acetonitrile (B) were used at a flow rate of 1 ml min^−1^ with the binary gradient conditions 50–70% B from 0 to 5 min, 70–92% B from 5 to 10 min, and 92–96% B from 10 to 28 min. For enzyme kinetics and characterization (HPLC method 2), 0.1% formic acid in water (A) and acetonitrile (B) were used at a flow rate of 1 ml min^−1^ with the binary gradient conditions 60–70% B from 0 to 5 min, 70–92% B from 5 to 7 min, and 92–42.5% B from 7 to 15 min.

For LC‐ESI‐HRMS analysis, a Vanquish UHPLC coupled to an Orbitrap Exploris 120 mass spectrometer (Thermo Scientific, Bremen, Germany) was used. Samples were run on a Poroshell 120 EC‐C18 column (4.6 × 100 mm, 2.7 μm) at a flow rate of 0.5 ml min^−1^ using 0.1% formic acid in water (A) and acetonitrile (B) with the binary gradient conditions 40–80% B from 0 to 4 min, and 80–100% B from 4 to 21 min. ESI‐HRMS data were obtained in positive or negative ionization modes using an H‐ESI‐Ion source with a static spray voltage of 2.5 kV. Sheath, aux, and sweep gases were applied at 50, 10, and 1 arbitrary units, respectively. The temperature of the ion transfer tube and the vaporizer were set at 325 and 350°C, respectively. The Orbitrap mass resolution for small molecules (scan range *m/z* 200–650) was set to 120 k. At the beginning of each run, EASY‐IC mass calibration was used for mass shift correction. Data acquisition and analysis were performed using the Xcalibur v4.6.67.17 software (Thermo Scientific, Bremen, Germany).

The amounts (ng) of products 4 and 5 were determined from their peak areas using standard curves. The percentage of each product was calculated as its proportion of the total amount of both products.

Samples for MS/MS analysis were directly infused into a 3200 QTrap mass spectrometer (Applied Biosystems/MDS SCIEX, Darmstadt, Germany), equipped with a Turbo V ion source (AB/MDS SCIEX) and a 2.3 mm syringe pump (Hamilton, Reno, USA). ESI‐MS data were obtained in positive or negative ionization modes, setting the source voltage to 4.5 kV and the declustering potential to 76 V. Nitrogen gas was used for nebulization. The pseudo molecular ion peaks obtained were further fragmented by collision‐induced dissociation (−15 to −40 eV, nitrogen gas). For data acquisition and processing, the Analyst v1.64.2 software (AB/MDS SCIEX) was used.

### Protein modeling

The 3D structural models of *Hs*RPThxa and *Hp*RPThxa were predicted using the AlphaFold2 software (Jumper et al., 2021). The quality of the generated models (Figure S21) was assessed by calculating various energetical, geometrical, and structural descriptors using QMEAN4, QMEANDisCO (Benkert et al., 2009), Energy Z score (Melo et al., 1997), MolProbity score, and Clash score (Davis et al., 2007) (Table S7). In total, five models each were evaluated in this manner. The final models were selected based on the best performance during the evaluation process. Additionally, coverage of models was checked, and the validity of secondary structure for the selected models was analyzed by comparing them to the secondary structure predictions for *Hs*RPThxa and *Hp*RPThxa sequences using different servers: PSIPRED (McGuffin et al., 2000), PSSPred (Yan et al., 2013), Jpred4 (Drozdetskiy et al., 2015), and NetSurfP‐3.0 server (Høie et al., 2022) (Figure S22). All models generated by AlphaFold2 appeared to predict an apparently disordered loop in the N‐terminal region between positions 1–95 in both the *Hs*RPThxa and *Hp*RPThxa enzymes. Additional analysis to define this region was performed to test whether the predicted disordered region was due to the sequence data or the lack of structural information. Secondary structure was determined based on the protein sequence using four different secondary structure prediction servers. A comparison of the predicted and modeled secondary structure for the *Hp*RPThxa sequence showed a high degree of resemblance, especially the existence of the disordered loop in the N‐terminal region (1–95). In addition, the N‐terminal region exhibits a low degree of evolutionary conservation and, arguably, concomitantly reduced biological significance, which was estimated using the Evolutionary Trace Server with default parameters (Lichtarge et al., 1996; Lua et al., 2015; Mihalek et al., 2004) (Figure S23). Although the global RMSD of the aligned models with the closest experimentally solved structure (4TQ5) is about 4.4 Å, the core part of the models is aligned very well (RMSD = 1.2 Å), and the active site geometry is highly similar to be used as a template for further docking simulations.

Based on the final apoenzyme models for *Hs*RPThxa and *Hp*RPThxa (Figure S24), a second holoenzyme model each was built with Mg^2+^ ions and the prenyl donor DMAPP. The conformation and orientation of the DMAPP chain in the crystal structure of *Archaeoglobus fulgidus* AfUbiA (PDB ID 4TQ3, chain A) served as the initial reference to dock DMAPP. This is because visual inspection showed that the active sites of AfUbiA and both *Hs*RPThxa and *Hp*RPThxa share a similar architecture (i.e., same or similar residues located in the same positions). We aligned the AfUbiA and *Hs*RPThxa and *Hp*RPThxa structures and dragged a GPP molecule into the active site of *Hs*RPThxa and *Hp*RPThxa each (align the diphosphate group in place to coordinate with the Mg^2+^ ions). Then, we modified GPP to DMAPP in PyMOL2 and the catalytically important conformation of DMAPP was optimized by docking in MOE 2022.2 (Chemical Computing Group, Montreal, Canada). The homology modeling for structure prediction was also performed using YASARA 23.5.19 (Krieger et al., 2002) to cross‐validate the AlphaFold2 predictions and to predict the binding pose of Mg^2+^ ions and DMAPP using PDB IDs 4TQ3, 4TQ5, and 6 M31. The generated holoenzyme models of *Hs*RPThxa and *Hp*RPThxa containing Mg^2+^ ions and DMAPP (Figure S25) were further energy‐minimized to remove subtle steric clashes and used for mechanism‐based docking of the acceptor substrate patulone 2.

### Mechanism‐based molecular docking of substrate

Molecular docking was performed using MOE 2022.02 (Chemical Computing Group, Montreal, Canada). The predicted reactivity of enzyme‐substrate complexes was examined by mechanism‐based substrate docking with scoring of catalytically competent docking poses based on the catalytic mechanism proposed for PTs (Luk et al., 2011; Luk & Tanner, 2009). The docking procedure was as follows: A ligand database was prepared containing patulone by drawing a 3D representation using the MOE builder tool, exporting it directly to the mdb file. Additionally, protein models for docking were prepared according to the MOE's standard “Quick Prep” protocol, firstly protonating the structure (using the Protonate 3D tool) and, secondly, energy‐minimizing it based on steepest descent using AMBER10/EHT forcefield and allowing an RMS gradient of 0.1 kcal mol^−1^ Å^−2^. Lastly, the binding site for docking was identified using the SiteFinder tool with default parameters.

Docking was performed using the “general” docking tool for the ligand database. “Triangle Matcher” was used for placement, and the “Induced Fit” method was used for the refinement of ligand poses, (allowing flexible sidechains during docking). At least 1000 docking poses were generated and scored based on London ΔG + GBVI/WSA ΔG values. Then, the fifty best‐scored poses were further analyzed for the selection of the final docking pose. For mechanism‐based docking of the substrate, the *Hp*RPThxa and *Hs*RPThxa models with ions and DMAPP were used as receptors.

Final docking poses for each substrate were selected based on proposed catalytic distance criteria for the PT reaction (Luk et al., 2011, Luk & Tanner, 2009). Additionally, the scoring of each pose for the respective structures was examined.

### *In silico* site‐saturation mutagenesis

The residues surrounding the prenyl donor or acceptor in the active site of *Hs*RPThxa (4.5 Å radius) were selected for *in silico* site saturation mutagenesis, which was conducted using MOE. In total, 25 residues were selected (Table S3). Mutagenesis was carried out on these residues using variations encompassing twenty standard amino acids (including self‐mutant as a positive control for comparison). The stability of each variant, as well as its affinity towards the prenyl donor and acceptor, were evaluated, and variants with dramatically decreased stability and/or affinity were discarded (Table S4). Further visualization of variants' conformers was performed to investigate the catalytic competent poses for either forward or reverse prenylation reaction.

### Phylogenetic analysis

The evolutionary relationship between the identified RPThxa enzymes and other plant aPTs was inferred by using the Maximum Likelihood method based on the Jones *et al*. w/freq. model (Jones et al., 1992). The bootstrap consensus tree inferred from 1000 replicates is taken to represent the evolutionary history of the sequences analyzed (Felsenstein, 1985). Branches corresponding to partitions reproduced in less than 50% bootstrap replicates are collapsed. The percentage of replicate trees in which the associated sequences clustered together in the bootstrap test (1000 replicates) is shown next to the branches (Felsenstein, 1985). Initial tree(s) for the heuristic search were obtained automatically by applying Neighbor‐Joining and BioNJ algorithms to a matrix of pairwise distances estimated using a JTT model, followed by selecting the topology with the superior log likelihood value. A discrete Gamma distribution was used to model evolutionary rate differences among sites (5 categories [+G, parameter = 2.6539]). The rate variation model allowed for some sites to be evolutionarily invariable ([+I], 1.0460% sites). The analysis involved 70 amino acid sequences (Table S5). The final dataset included a total of 478 positions. Evolutionary analyses were conducted in MEGA7 (Kumar et al., 2016).

### Subcellular localization

A modified version of the binary plant expression vector pCambia 2300u, designed for translational fusion of a YFP reporter to the C‐terminus of a protein of interest (Nour‐Eldin et al., 2006), was used for localization experiments in *N. benthamiana* leaves. Transgenic leaf tissue was visualized 4 days post infiltration by laser scanning microscopy. The scan head of a cLSM‐510META confocal laser‐scanning microscope (Zeiss, Göttingen, Germany) was connected to an Axiovert 200 M equipped with Plan‐Neofluar 10 × 0.3^−1^ and C‐Apochromat 40 × 1.2^−1^ water‐immersion objectives. A 488 nm argon laser was used for fluorescence excitation. Images were recorded using ZEN 2009 software (Zeiss, Göttingen, Germany).

## ACCESSION NUMBERS

The sequences of the cloned genes were deposited in the NCBI GenBank as follows: *HpRPThxa* (PP836386), *HsRPThxa* (PP836387).

## AUTHOR CONTRIBUTIONS

LE, HMBS, LB, BL, IE‐A designed the research. LE, HMBS, BL, IE‐A analyzed transcriptomic data. HMBS cloned, expressed, and functionally characterized *Hp*RPThxa in *S. cerevisiae*. LE, TM cloned, expressed, and functionally characterized *Hs*RPThxa in *S. cerevisiae*. LE, HL extracted, purified, and identified the isolated compounds and performed NMR experiments. LE performed leaf disk assays and subcellular localization in *N. benthamiana*. AH and MD performed molecular modeling and docking and wrote the respective sections. RM, DK provided guidance and assisted in subcellular localization experiments. RM performed *in vivo* production in yeast and wrote the respective section. HMBS, IE‐A carried out phylogenetic analysis and synthesized acceptor and donor substrates. LE, HMBS, LB, IE‐A wrote the manuscript with input from coauthors. All authors read and approved the final manuscript.

## CONFLICT OF INTEREST

The authors declare no competing interests.

## Supporting information


**Figure S1.** Types of prenylation reactions and examples of soluble microbial enzymes involved.
**Figure S2.** Bioactive reverse‐prenylated phenolics.
**Figure S3.** Chemical shifts of isolated hyperixanthone A 4.
**Figure S4.** Chemical shifts of isolated allanxanthone C 5.
**Figure S5.** ^1^H spectrum of hyperixanthone A 4.
**Figure S6.** ^13^C spectrum of hyperixanthone A 4.
**Figure S7.** ^1^H–^1^HCOSY spectrum of hyperixanthone A 4.
**Figure S8.** Superimposed ^1^H–^13^C HSQC and ^1^H–^13^C HMBC spectra of hyperixanthone A 4.
**Figure S9.** ^1^H spectrum of allanxanthone C 5.
**Figure S10.** DEPTQ spectrum of allanxanthone C 5.
**Figure S11.** Superimposed ^1^H–^13^C HSQC and ^1^H–^13^C HMBC spectra of allanxanthone C 5.
**Figure S12.** Extended xanthone profiles of *H. sampsonii* and *H. perforatum* root extracts.
**Figure S13.** Compounds screened as potential prenyl acceptors.
**Figure S14.** Characterization of *Hs*RPThxa *in vitro*.
**Figure S15.** Characterization of *Hp*RPThxa *in vitro*.
**Figure S16.** Michaelis–Menten kinetics of *Hs*RPThxa, *Hs*RPThxa‐tr and *Hp*RPThxa.
**Figure S17.** Concerted activity of *H. sampsonii* aPTs.
**Figure S18.** Substrate docking poses in the cavity of two inactive *Hs*RPThxa variants.
**Figure S19.** Proposed biosynthetic pathway of polyprenylated xanthones identified in *H. perforatum* and *H. sampsonii*.
**Figure S20.** Examples of reverse‐prenylated xanthones previously reported from various *Hypericum* species.
**Figure S21.** Multiple structural alignment of 3D models.
**Figure S22.** Q3 secondary structure prediction for *Hp*RPThxa and *Hs*RPThxa sequences.
**Figure S23.** Estimation of biological importance of *Hs*RPThxa amino acid residues.
**Figure S24.** Alignment of *Hp*RPThxa and *Hs*RPThxa models with the crystal structure of UBIAD1.
**Figure S25.** Holoenzyme models of *Hs*RPThxa and *Hp*RPThxa showing the interaction with magnesium ions and DMAPP.
**Table S1.** Expression data for candidate aPTs from *H. sampsonii* (*H.s*.) transcriptomes and corresponding FPKM values for homologous aPTs from *H. perforatum*.
**Table S2.** Kinetic parameters of *Hp*RPThxa, HsRPThxa and *Hs*RPThxa‐tr.
**Table S3.** List of the selected residues for *in silico* site saturated mutagenesis.
**Table S4.** Changes in stability and affinity of the selected mutants from *in silico* site saturated mutagenesis.
**Table S5.** Accession numbers of the aPT sequences used to construct the phylogenetic tree.
**Table S6.** Primer sequences.
**Table S7.** Evaluation of the 3D models predicted by the AlphaFold2 program.

**Data S1.** *In silico* site saturated mutagenesis.

## Data Availability

The data associated with this article are available in the article and in its online supporting information.
