# Supplementary material for: Reverse prenylation in plants by non‐canonical aromatic prenyltransferases
Source: Plant J. 2025 Jun 17;122(6):e70268. doi: 10.1111/tpj.70268 (PMC12172877; doi:10.1111/tpj.70268)
Supplement: Supplementary file 1 — Figure S1. Types of prenylation reactions and examples of soluble microbial enzymes involved. Figure S2. Bioactive reverse‐prenylated phenolics. Figure S3. Chemical shifts of isolated hyperixanthone A 4. Figure S4. Chemical shifts of isolated allanxanthone C 5. Figure S5. 1H spectrum of hyperixanthone A 4. Figure S6. 13C spectrum of hyperixanthone A 4. Figure S7. 1H–1HCOSY spectrum of hyperixanthone A 4. Figure S8. Superimposed 1H–13C HSQC and 1H–13C HMBC spectra of hyperixanthone A 4. Figure S9. 1H spectrum of allanxanthone C 5. Figure S10. DEPTQ spectrum of allanxanthone C 5. Figure S11. Superimposed 1H–13C HSQC and 1H–13C HMBC spectra of allanxanthone C 5. Figure S12. Extended xanthone profiles of H. sampsonii and H. perforatum root extracts. Figure S13. Compounds screened as potential prenyl acceptors. Figure S14. Characterization of HsRPThxa in vitro. Figure S15. Characterization of HpRPThxa in vitro. Figure S16. Michaelis–Menten kinetics of HsRPThxa, HsRPThxa‐tr and HpRPThxa. Figure S17. Concerted activity of H. sampsonii aPTs. Figure S18. Substrate docking poses in the cavity of two inactive HsRPThxa variants. Figure S19. Proposed biosynthetic pathway of polyprenylated xanthones identified in H. perforatum and H. sampsonii. Figure S20. Examples of reverse‐prenylated xanthones previously reported from various Hypericum species. Figure S21. Multiple structural alignment of 3D models. Figure S22. Q3 secondary structure prediction for HpRPThxa and HsRPThxa sequences. Figure S23. Estimation of biological importance of HsRPThxa amino acid residues. Figure S24. Alignment of HpRPThxa and HsRPThxa models with the crystal structure of UBIAD1. Figure S25. Holoenzyme models of HsRPThxa and HpRPThxa showing the interaction with magnesium ions and DMAPP. Table S1. Expression data for candidate aPTs from H. sampsonii (H.s.) transcriptomes and corresponding FPKM values for homologous aPTs from H. perforatum. Table S2. Kinetic parameters of HpRPThxa, HsRPThxa and HsRPThxa‐tr. [file TPJ-122-0-s001.pdf]

# SUPPORTING INFORMATION

# Reverse prenylation in plants by non-canonical aromatic prenyltransferases

Lukas Ernst[1,2,#], Hesham MB Sayed[1,3,#], Ahmed Hassanin[3,4], Rebekka Moegenburg[1,2], Tomke Meents[1,2], Hui Lyu[5], David Kaufholdt[6], Mehdi D Davari[4], Ludger Beerhues[1,2], Benye Liu[1,2]*, Islam El-Awaad[1,2,3]*

[1]Technische Universität Braunschweig, Institute of Pharmaceutical Biology, Mendelssohnstraße 1, 38106 Braunschweig, Germany; [2]Technische Universität Braunschweig, Center of Pharmaceutical Engineering (PVZ), Franz-Liszt-Straße 35 A, 38106 Braunschweig, Germany; [3]Assiut University, Faculty of Pharmacy, Department of Pharmacognosy, 71526, Egypt; [4] Leibniz Institute of Plant Biochemistry, Department of Bioorganic Chemistry, Weinberg 3, 06120 Halle (Saale), Germany; [5]Max Planck Institute for Chemical Ecology, NMR/Biosynthesis Group, Jena 07745, Germany; [6]Technische Universität Braunschweig, Institute of Plant Biology, Humboldtstraße 1, 38106 Braunschweig, Germany

*Corresponding authors. Email: islam.elawaad@tu-braunschweig.de, b.liu@tu-braunschweig.de

[#]These authors contributed equally to this work.

## Table of Contents

**Supporting Figures** ... **3**
**Figure S1.** Types of prenylation reactions and examples of soluble microbial enzymes involved ... 3
**Figure S2.** Bioactive reverse-prenylated phenolics ... 4
**Figure S3.** Chemical shifts of isolated hyperixanthone A **4** ... 5
**Figure S4.** Chemical shifts of isolated allanxanthone C **5** ... 5
**Figure S5.** $^{1}H$ spectrum of hyperixanthone A **4** ... 6
**Figure S6.** $^{13}C$ spectrum of hyperixanthone A **4** ... 6
**Figure S7.** $^{1}H$–$^{1}H$COSY spectrum of hyperixanthone A **4** ... 7
**Figure S8.** Superimposed $^{1}H$–$^{13}C$ HSQC and $^{1}H$–$^{13}C$ HMBC spectra of hyperixanthone A **4** ... 7
**Figure S9.** $^{1}H$ spectrum of allanxanthone C **5** ... 8
**Figure S10.** DEPTQ spectrum of allanxanthone C **5** ... 8
**Figure S11.** Superimposed $^{1}H$–$^{13}C$ HSQC and $^{1}H$–$^{13}C$ HMBC spectra of allanxanthone C **5** ... 9
**Figure S12.** Extended xanthone profiles of *H. sampsonii* and *H. perforatum* root extracts ... 10
**Figure S13.** Compounds screened as potential prenyl acceptors ... 11
**Figure S14.** Characterization of *Hs*RPThxa *in vitro* ... 12
**Figure S15.** Characterization of *Hp*RPThxa *in vitro* ... 13
**Figure S16.** Michaelis-Menten kinetics of *Hs*RPThxa, *Hs*RPThxa-tr and *Hp*RPThxa ... 14
**Figure S17.** Concerted activity of *H. sampsonii* aPTs ... 15
**Figure S18.** Substrate docking poses in the cavity of two inactive *Hs*RPThxa variants ... 16
**Figure S19.** Proposed biosynthetic pathway of the identified polyprenylated xanthones. ... 17
**Figure S20.** Examples of reverse prenylated xanthones previously reported from various *Hypericum* species ... 17
**Figure S21.** Multiple structural alignment of 3D models ... 18
**Figure S22.** Q3 secondary structure prediction for *Hp*RPThxa and *Hs*RPThxa sequences ... 19
**Figure S23.** Estimation of biological importance of *Hs*RPThxa amino acid residues ... 20
**Figure S24.** Alignment of *Hp*RPThxa and *Hs*RPThxa models with the crystal structure of UBIAD1 20
**Figure S25.** Holoenzyme models of *Hs*RPThxa and *Hp*RPThxa ... 21

**Supporting Tables** ... **22**
**Table S1.** Expression data for candidate aPTs from *H. sampsonii* transcriptomes and corresponding FPKM values for homologous aPTs from *H. perforatum* ... 22
**Table S2.** Kinetic parameters of *Hp*RPThxa, *Hs*RPThxa and *Hs*RPThxa-tr ... 23
**Table S3.** List of the selected residues for *in silico* site saturated mutagenesis ... 24
**Table S4.** Changes in stability and affinity of the selected mutants ... 24
**Table S5.** Accession numbers of the aPTs sequences used to construct the phylogenetic tree ... 25
**Table S6.** Primer sequences ... 27
**Table S7.** Evaluation of the 3D models predicted by AlphaFold2 ... 27

**Supporting References** ... **27**

## Supporting Figures

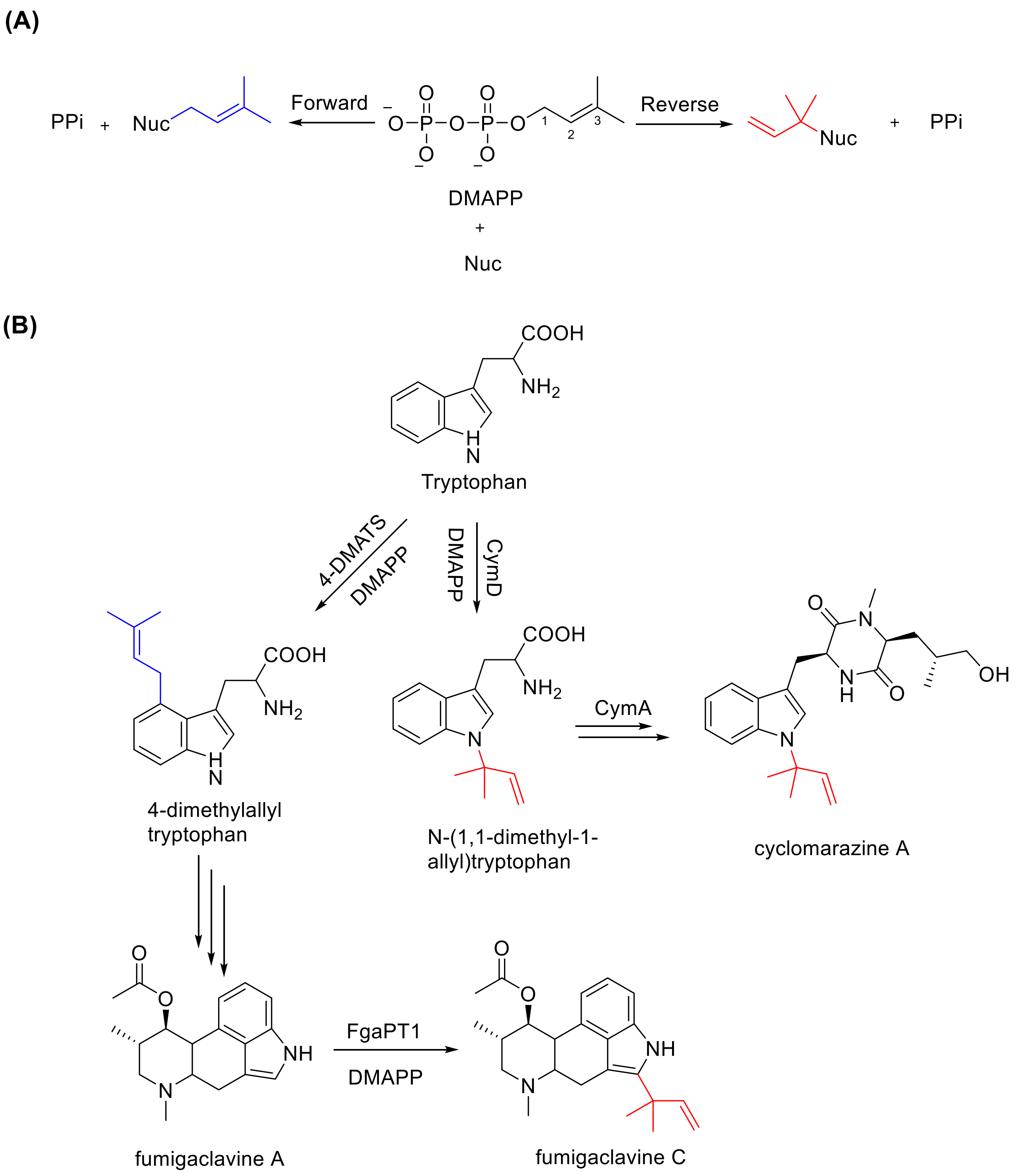


**Figure S1.** Types of prenylation reactions and examples of soluble microbial enzymes involved. (A) Forward and reverse prenylation reactions. DMAPP, dimethylallyl pyrophosphate. Nuc, nucleophilic oxygen, nitrogen, or carbon of the prenyl acceptor. (B) Representative examples of normal and reverse prenylations catalyzed by soluble microbial aPTs. Forward prenyl groups are depicted in blue while reverse prenyl groups are highlighted in red.

**(A)**

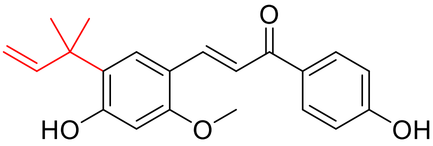

licochalcone A
(chalcone; antiinflammatory)

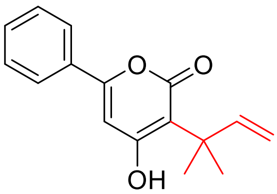

peplidiforone A
(phenylpyrone; antifungal)

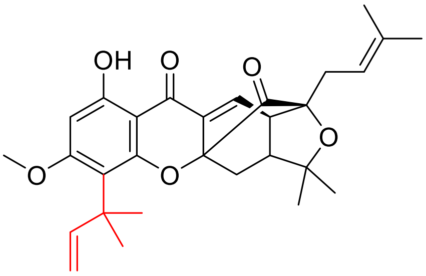

neobractatin
(phloroglucinol; anticancer)

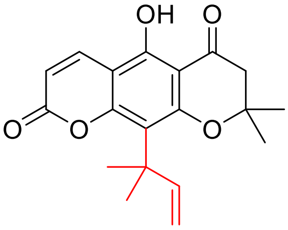

clausenidin
(coumarin; antiviral)

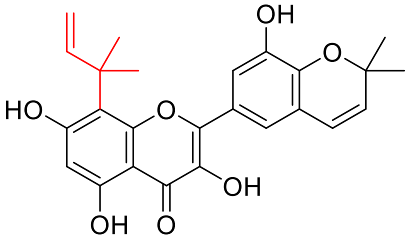

broussonol A
(flavonol; cytotoxic)

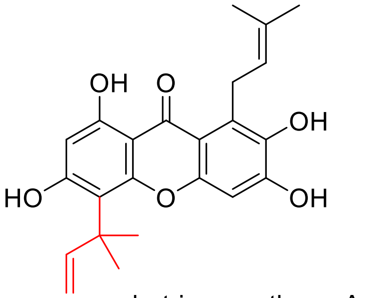

cudratricusxanthone A
(xanthone; antiinflammatory)

**(B)**

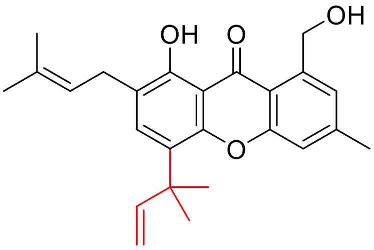

staprexanthone E
antidiabetic
*Stachybotrys chartarum*
Stachybotryaceae

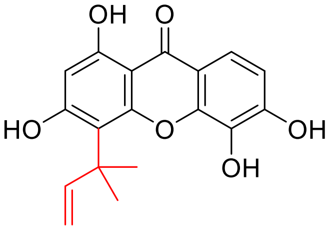

isocudraniaxanthone A
antimalarial
*Garcinia vieillardii*
Clusiaceae

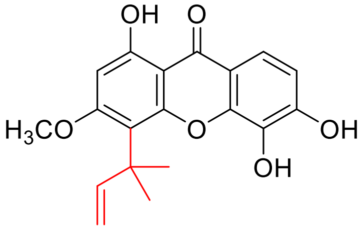

isocudraniaxanthone B
antimalarial
*Garcinia vieillardii*
Clusiaceae

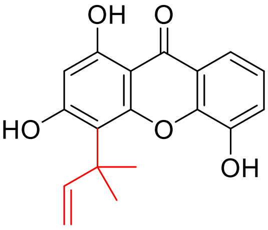

pancixanthone A
antimalarial
*Garcinia vieillardii*
Clusiaceae

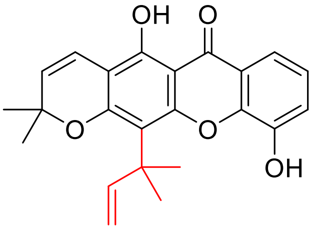

caloxanthone C
antibacterial
*Cudrania cochinchinensis*
Moraceae

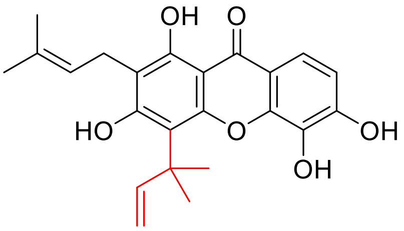

gerontoxanthone I
antibacterial
*Cudrania cochinchinensis*
Moraceae

**Figure S2.** Bioactive reverse-prenylated phenolics. (A) Examples of various classes of phenolic specialized metabolites, which carry a reverse prenyl group (depicted in red) and exhibit interesting pharmacological activities. (B) Examples of fungal and plant xanthones with a reverse prenyl group (red), which exhibit interesting biological activities.

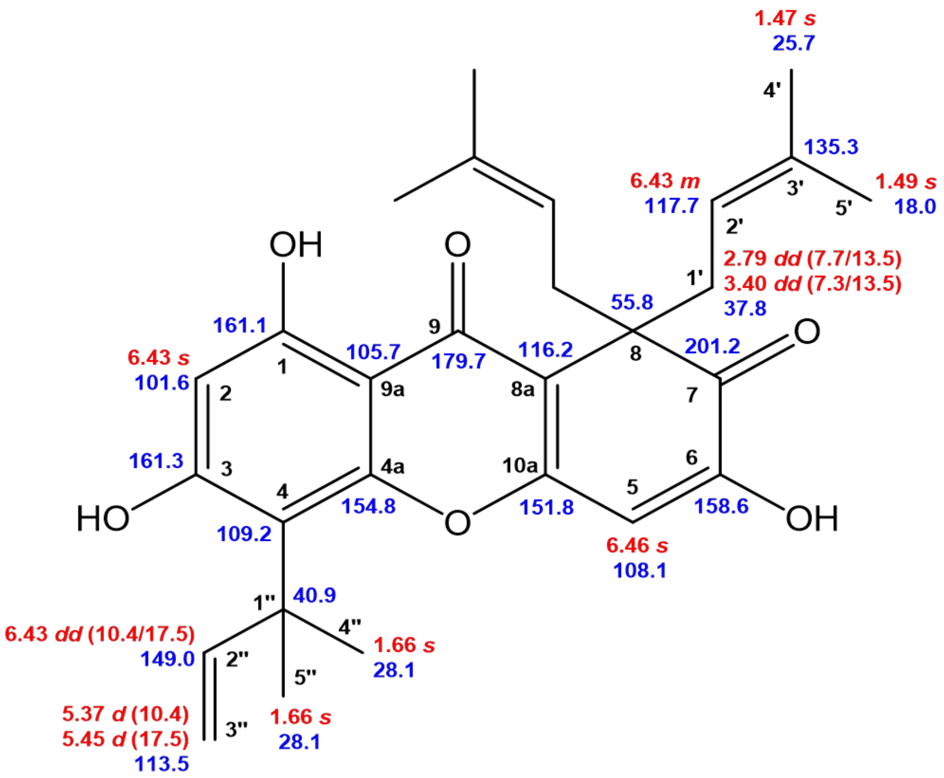

**Figure S3.** Chemical shifts of isolated hyperixanthone A **4**. Red: [1]H chemical shifts (δ ppm, *mult*., $^3J_{HH}$ in Hz). Blue: [13]C chemical shifts (δ ppm). Measured in $CDCl_3$.

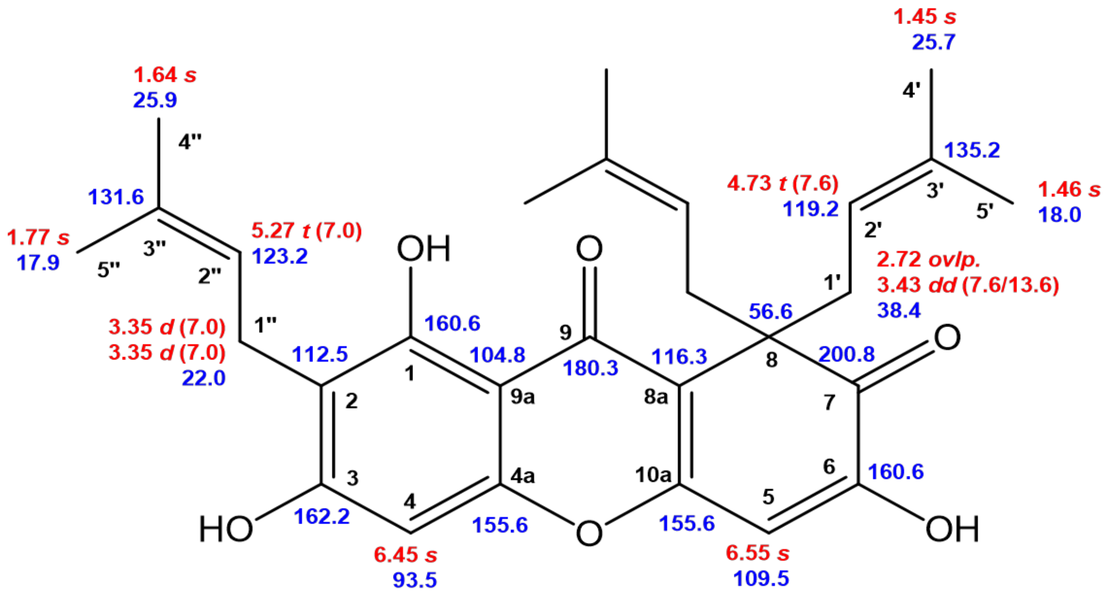

**Figure S4.** Chemical shifts of isolated allanxanthone C **5**. Red: [1]H chemical shifts (δ ppm, *mult*., $^3J_{HH}$ in Hz). Blue: [13]C chemical shifts (δ ppm). Measured in $C_3D_6O$.ovlp, overlapped with solvent signals.

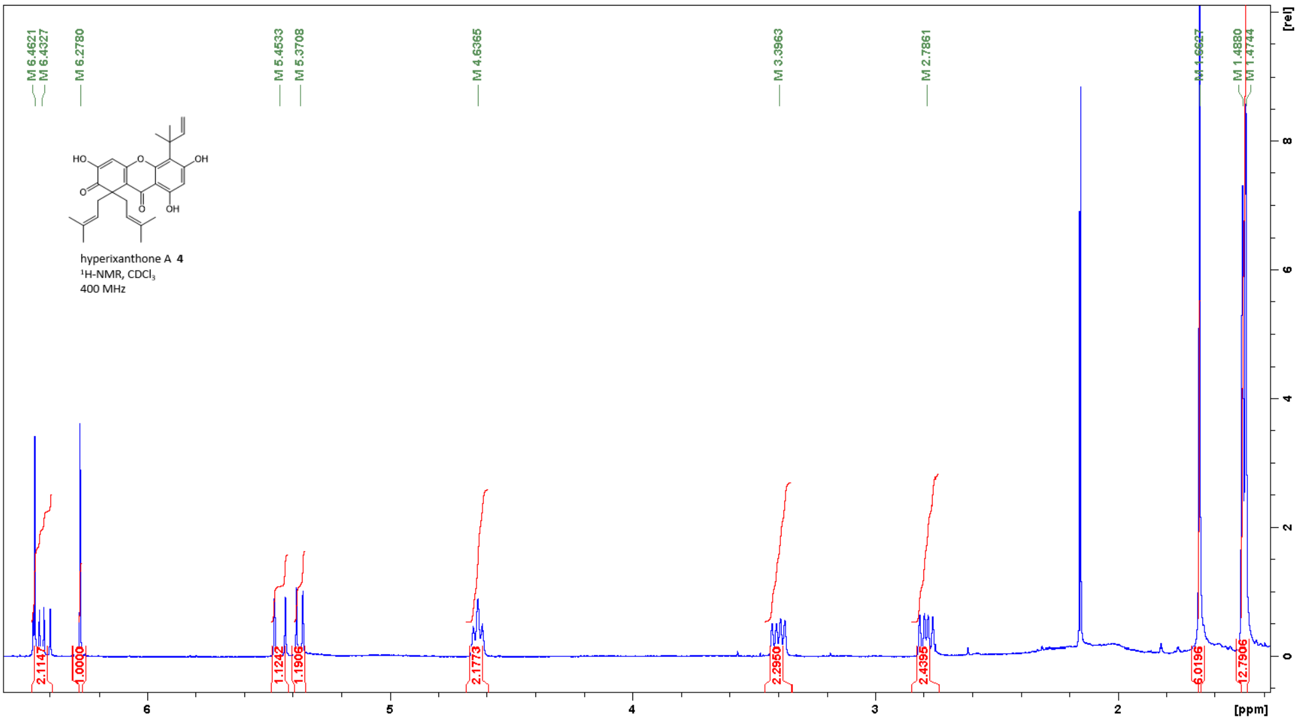


**Figure S5.** $^1$H spectrum of hyperixanthone A **4**. Measured in $CDCl_3$.

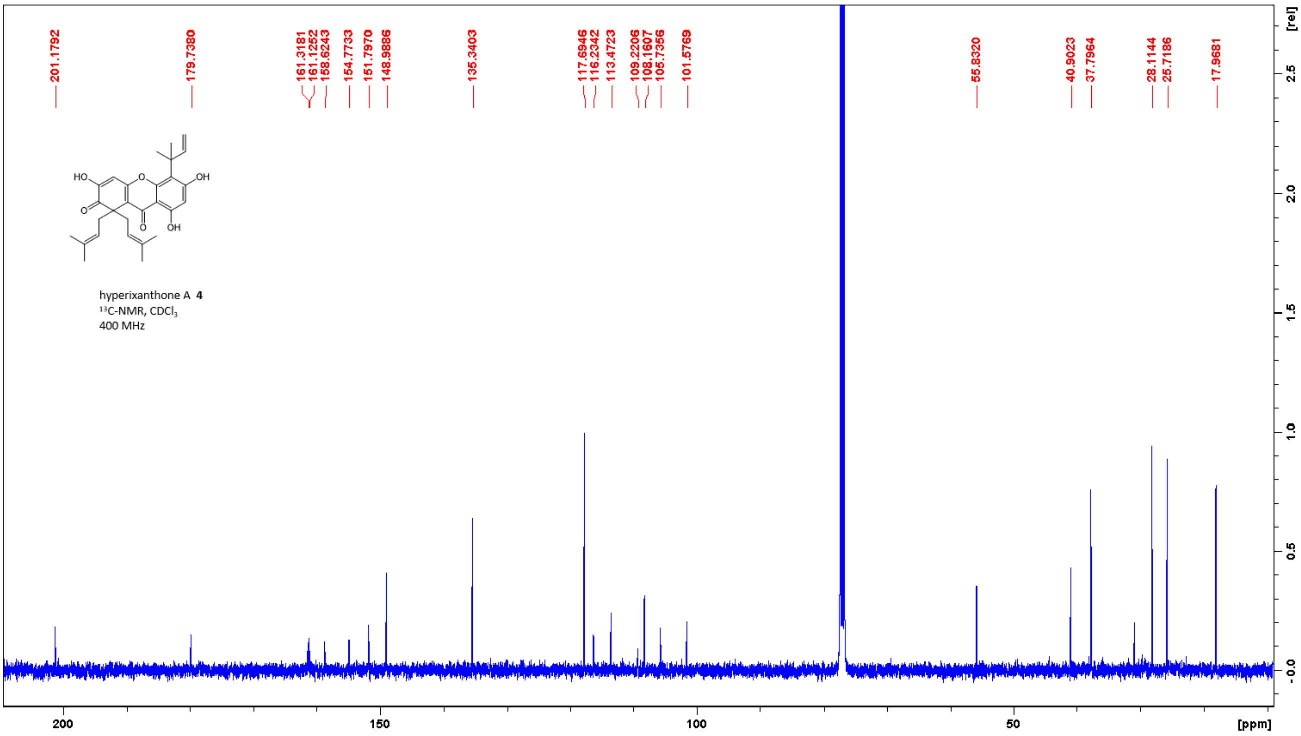


**Figure S6.** $^{13}$C spectrum of hyperixanthone A **4**. Measured in $CDCl_3$.

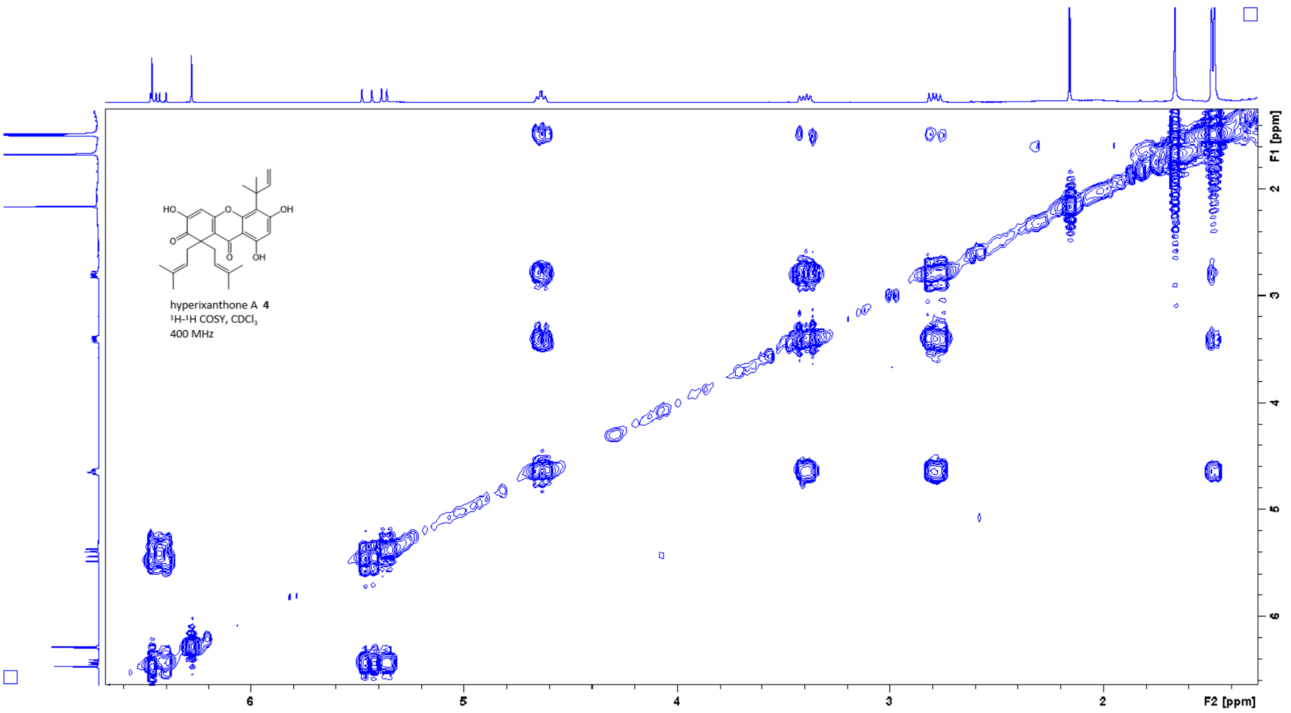


**Figure S7.** $^{1}$H–$^{1}$HCOSY spectrum of hyperixanthone A **4**. Measured in CDCl3.

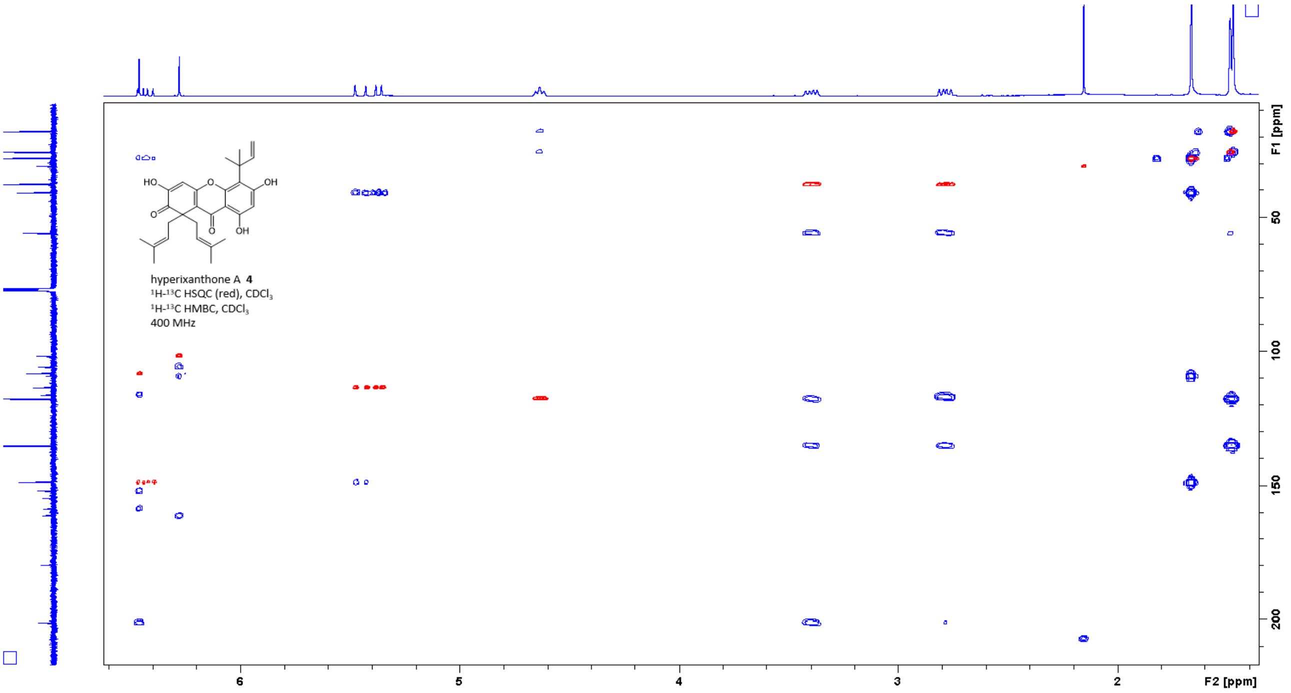


**Figure S8.** Superimposed $^{1}$H–$^{13}$C HSQC and $^{1}$H–$^{13}$C HMBC spectra of hyperixanthone A **4**. Measured in CDCl3.

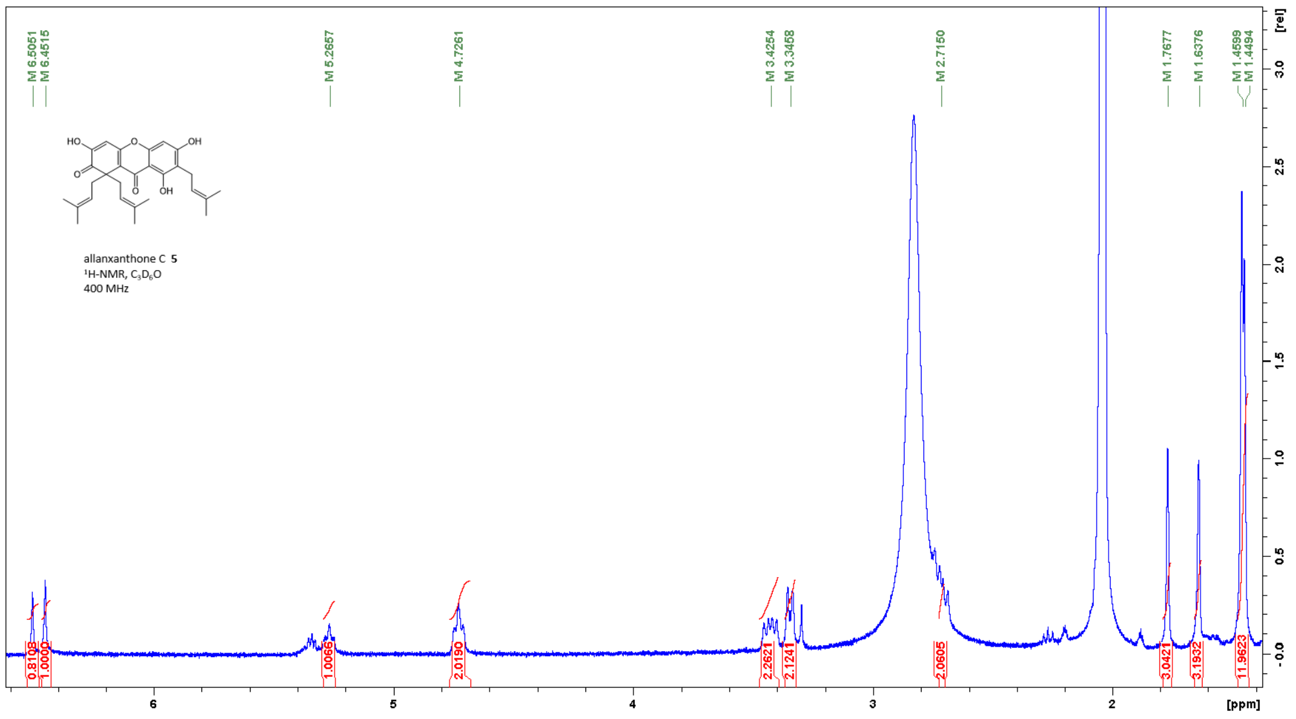


**Figure S9.** $^{1}$H spectrum of allanxanthone C **5**. Measured in $C_3D_6O$.

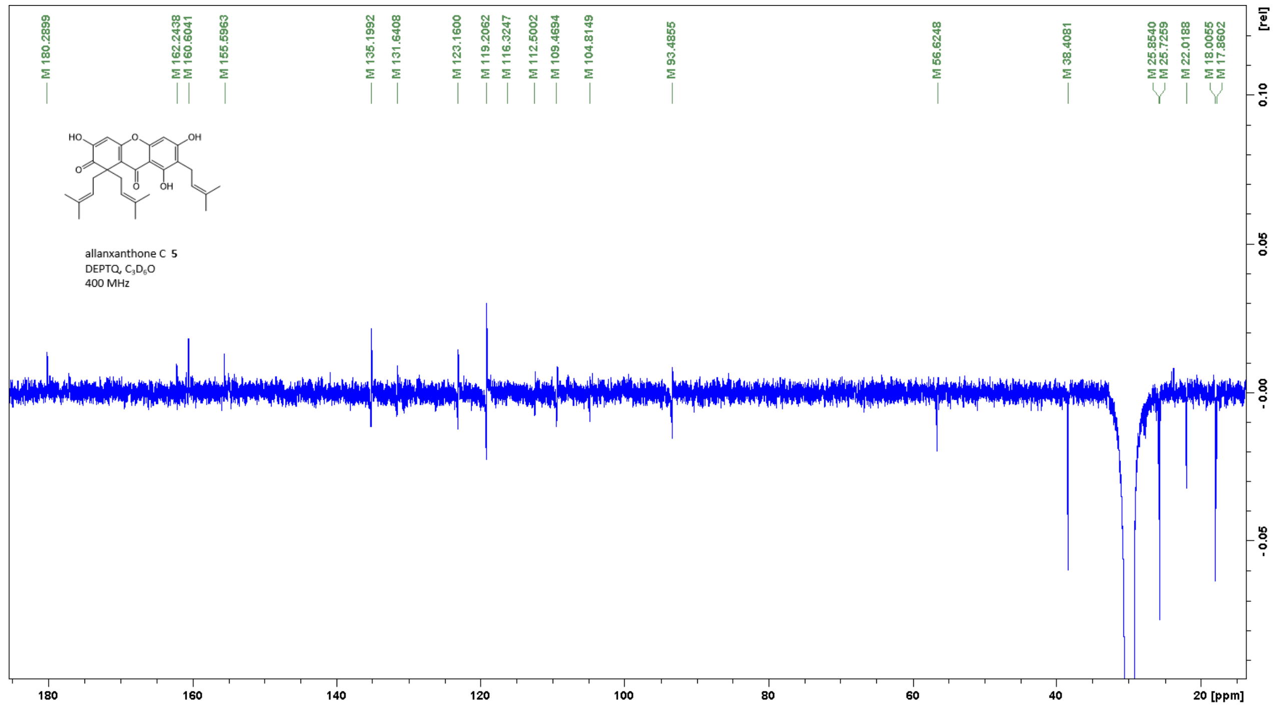


**Figure S10.** DEPTQ spectrum of allanxanthone C **5**. Measured in $C_3D_6O$.

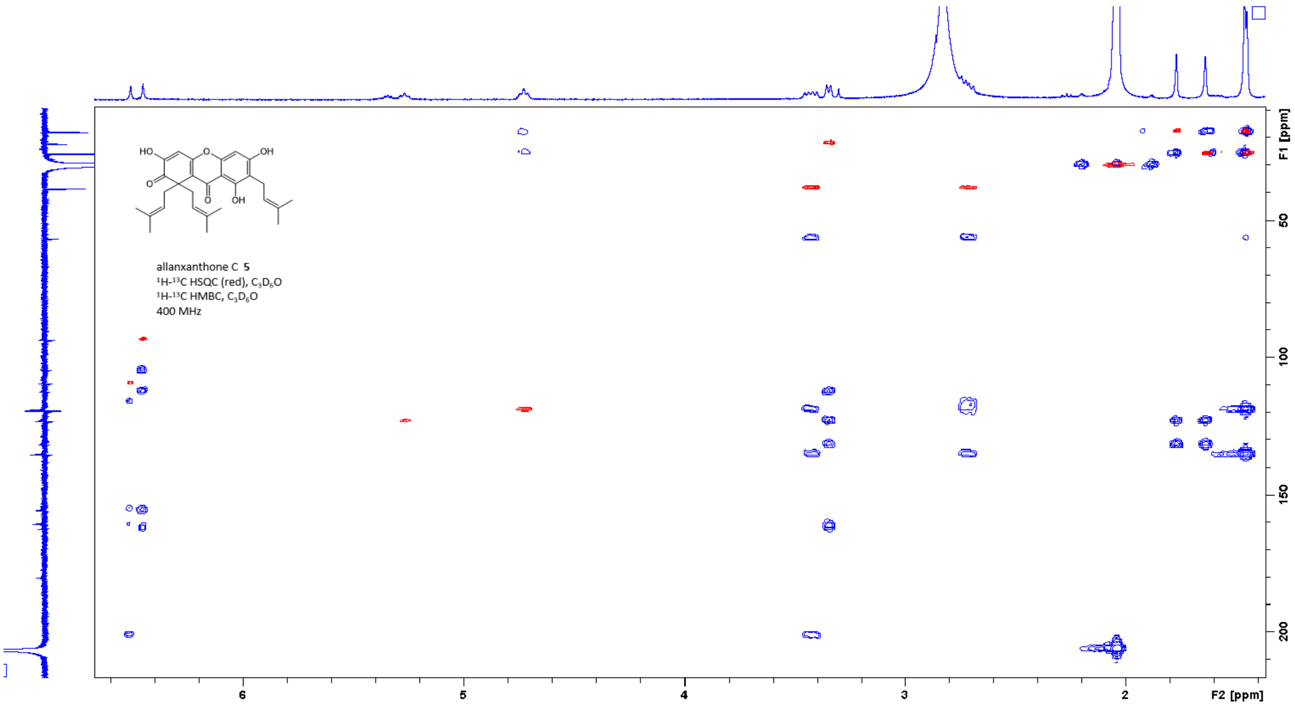

**Figure S11.** Superimposed $^{1}H$–$^{13}C$ HSQC and $^{1}H$–$^{13}C$ HMBC spectra of allanxanthone C **5**. Measured in $C_3D_6O$.

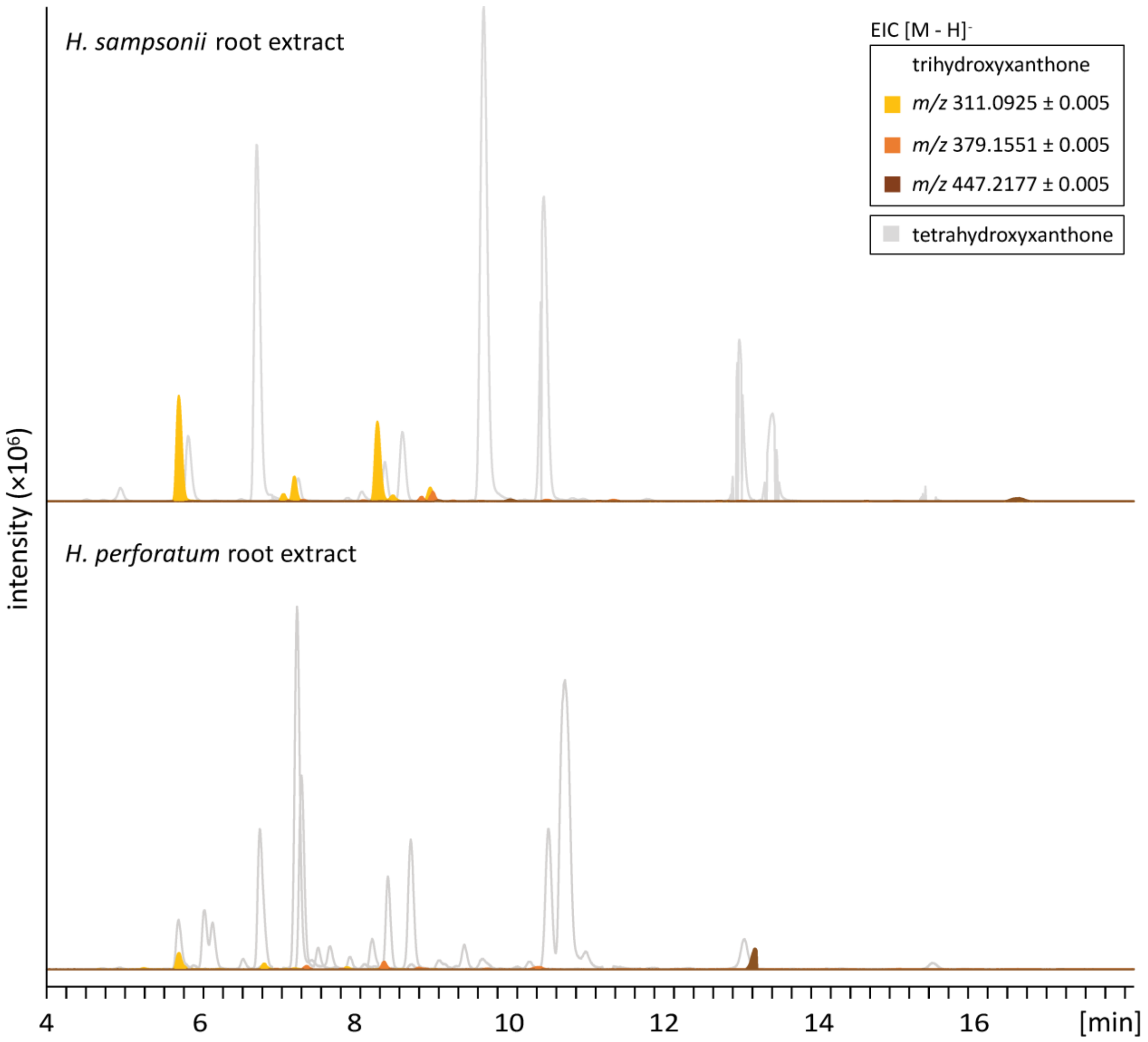


**Figure S12.** Extended xanthone profiles of *H. sampsonii* and *H. perforatum* root extracts. Extracted-ion chromatograms corresponding to the masses of mono- (*m/z* 311.0925), di- (*m/z* 379.1551), and triprenylated (*m/z* 447.2177) trihydroxyxanthones in *H. sampsonii* and *H. perforatum* root extracts. Only minor compounds were detected in comparison to the major signals of tetrahydroxylated constituents (grey).

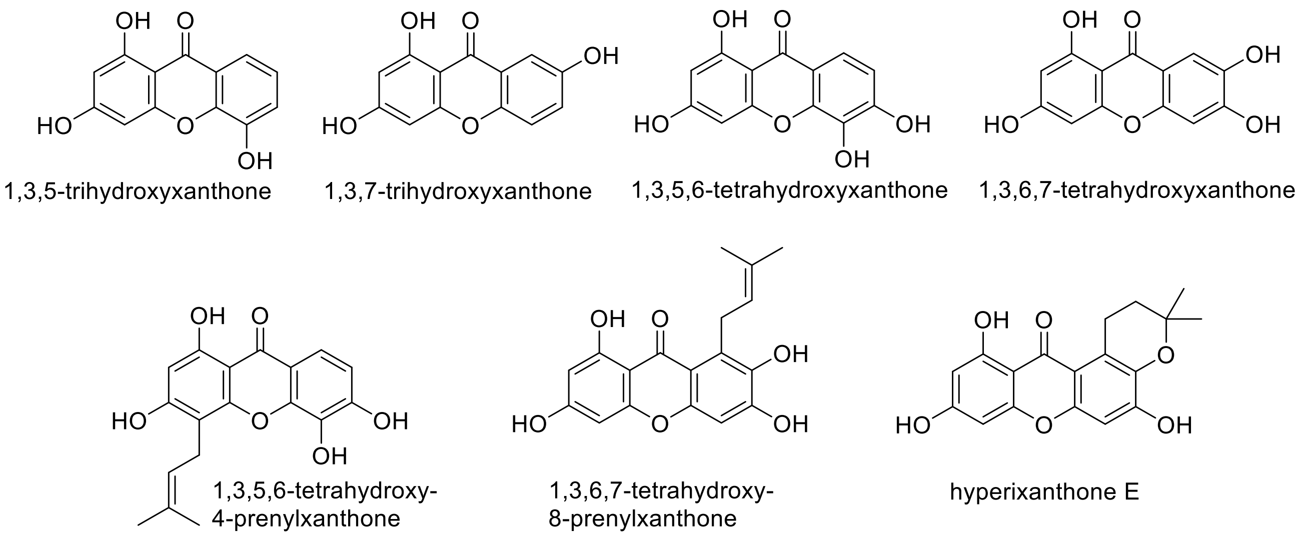


**Figure S13.** Compounds screened as potential prenyl acceptors to determine the specificity of RPThxa enzymes.

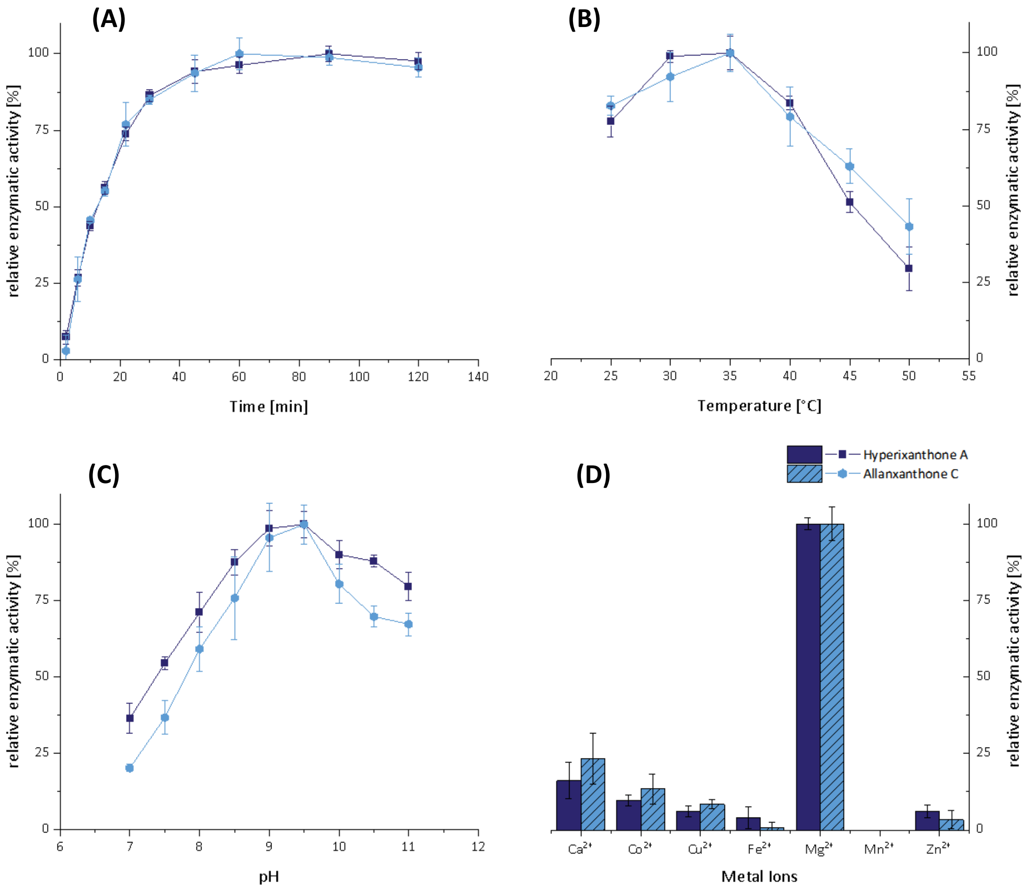


**Figure S14.** Characterization of *Hs*RPThxa *in vitro*. (A) The incubation time within the linear range was set to 8 min. (B) The optimal temperature was 30 - 35 °C. (C) The pH optimum was 9.0 - 9.5. (D) The preferred divalent metal ion was $Mg^{2+}$. Data represent means ± SD (n=3).

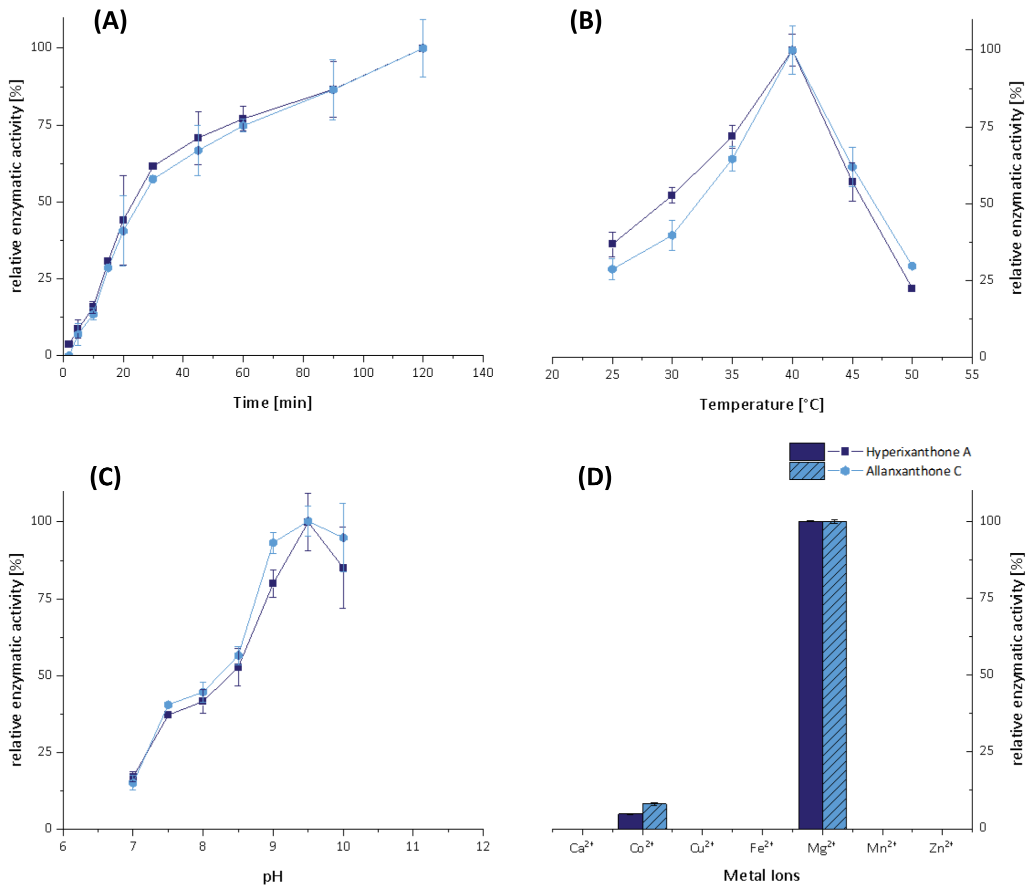


**Figure S15.** Characterization of *Hp*RPThxa *in vitro*. (A) The incubation time within the linear range was set to 15 minutes. (B) The optimal temperature was 40 °C. (C) The pH optimum was 9.5. (D) The preferred divalent metal ion was $Mg^{2+}$. Data represent means ± SD (n=3).

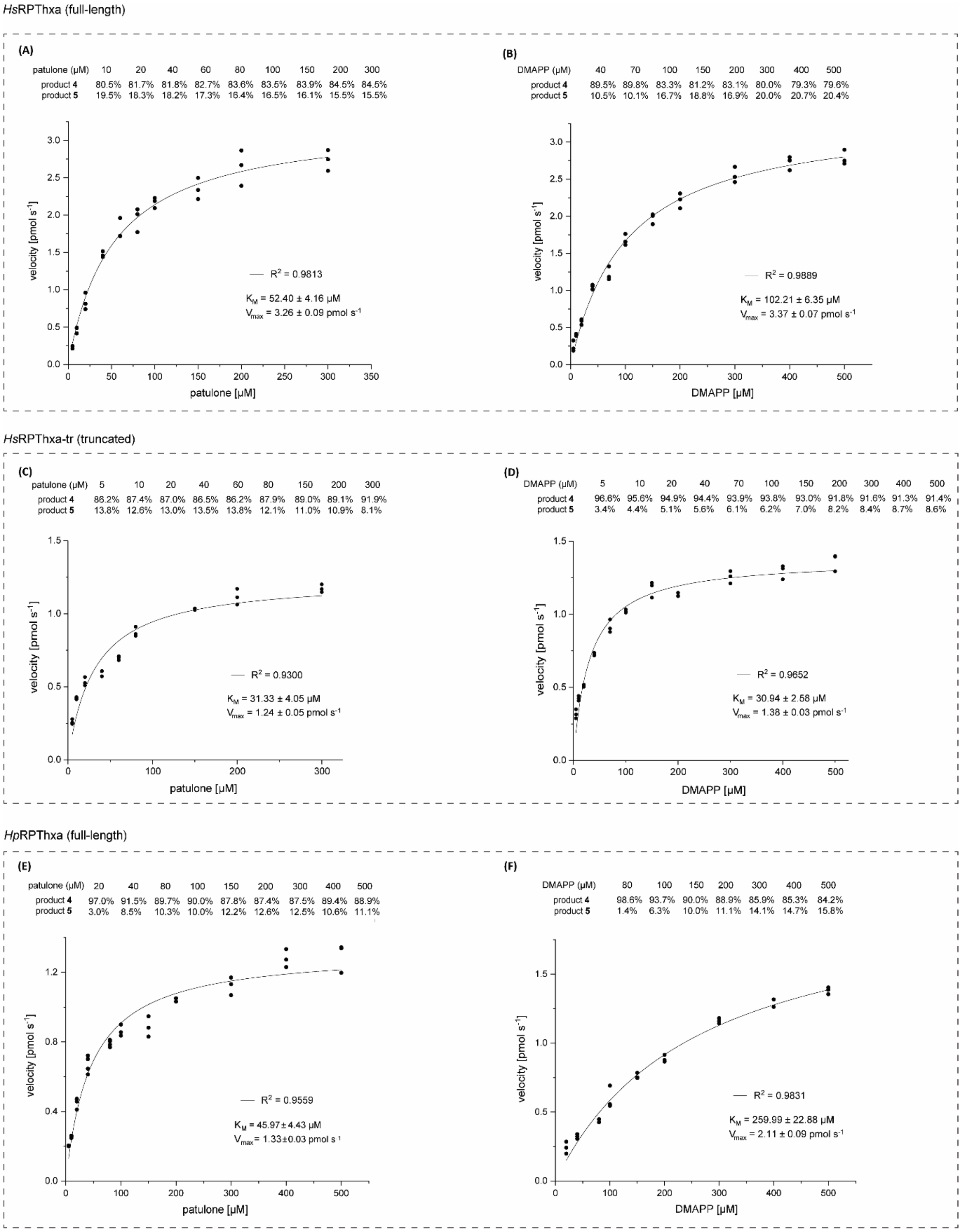


**Figure S16.** Michaelis-Menten kinetics of *Hs*RPThxa, *Hs*RPThxa-tr, and *Hp*RPThxa, determined for the reverse prenylation reaction with acceptor substrate **2** (A,C,E) and donor substrate DMAPP (B,D,F). The velocity of product **4** formation is plotted against increasing substrate concentrations (5–500 µM). The percentage ratios of the main product **4** and side product **5** are indicated above each curve at concentrations where product **5** was detectable. Increasing the concentration of patulone slightly increased the percentage of the major product **4** for the full-length and the truncated *Hs*RPThxa but led to a decrease in the percentage of **4** in the case of *Hp*RPThxa. Increasing the concentration of DMAPP decreased the percentage of **4** for all variants. The kinetic parameters for the acceptor substrate were determined at DMAPP saturation and vice versa. $K_M$, Michaelis-Menten constant; $V_{max}$, maximum velocity. Data represent means ± SD (n=3).

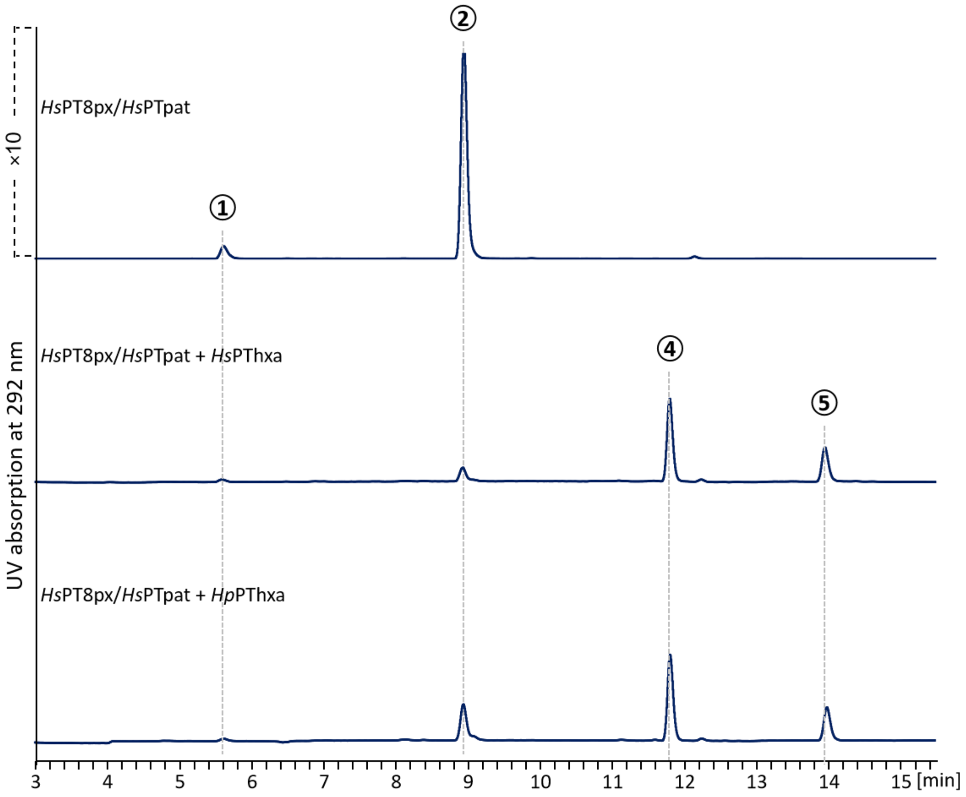


**Figure S17.** Concerted activity of *H. sampsonii* aPTs. HPLC-DAD analysis of enzyme assays containing microsomes of yeast cells co-transformed with *Hs*PT8px and *Hs*PTpat confirmed the efficient conversion of 1367THX to compound **2**. Additional co-transformation with *Hs*PThxa or *Hp*PThxa resulted in the formation of products **4** and **5**.

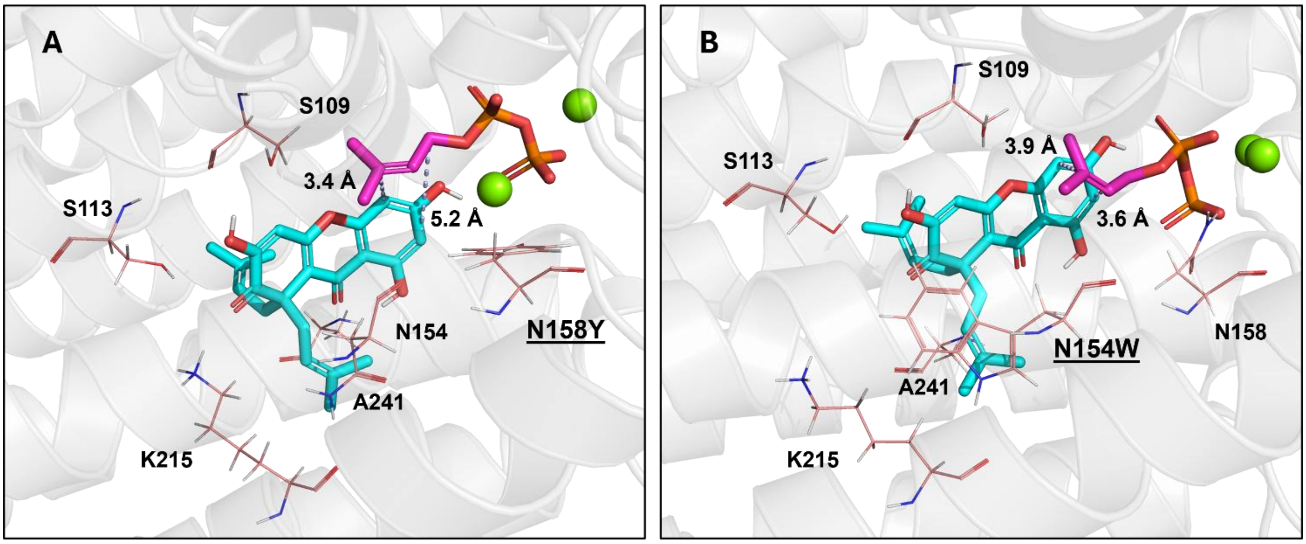


**Figure S18.** Substrate docking poses in the cavity of two inactive *Hs*RPThxa variants. (A) The catalytic docking pose of the N158Y variant shows favored distance for C-4 reverse over C-2 forward prenylation (B) The catalytic docking pose of the N154W variant shows almost equal distances for C-2 forward or C-4 reverse prenylations.

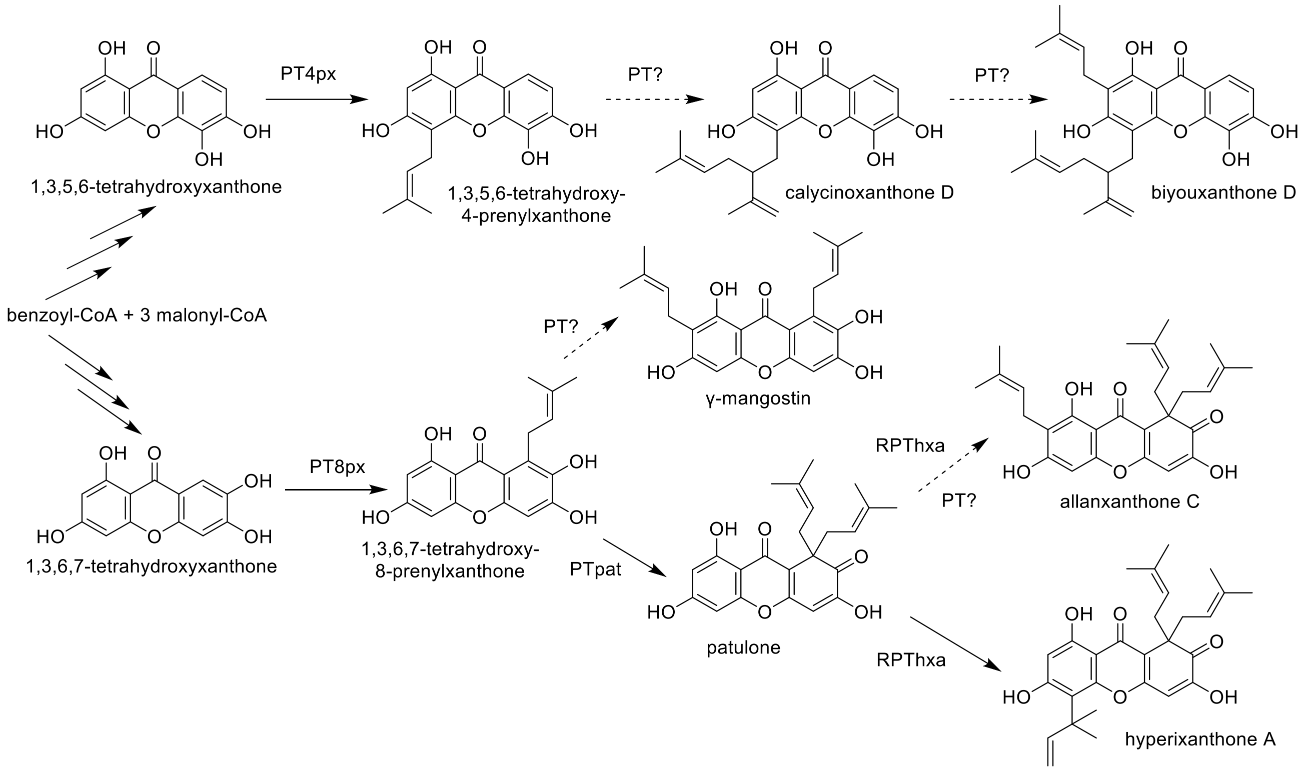


**Figure S19.** Proposed biosynthetic pathway of polyprenylated xanthones identified in *H. perforatum* and *H. sampsonii*. Dashed arrows refer to reactions catalyzed by yet unidentified PTs.

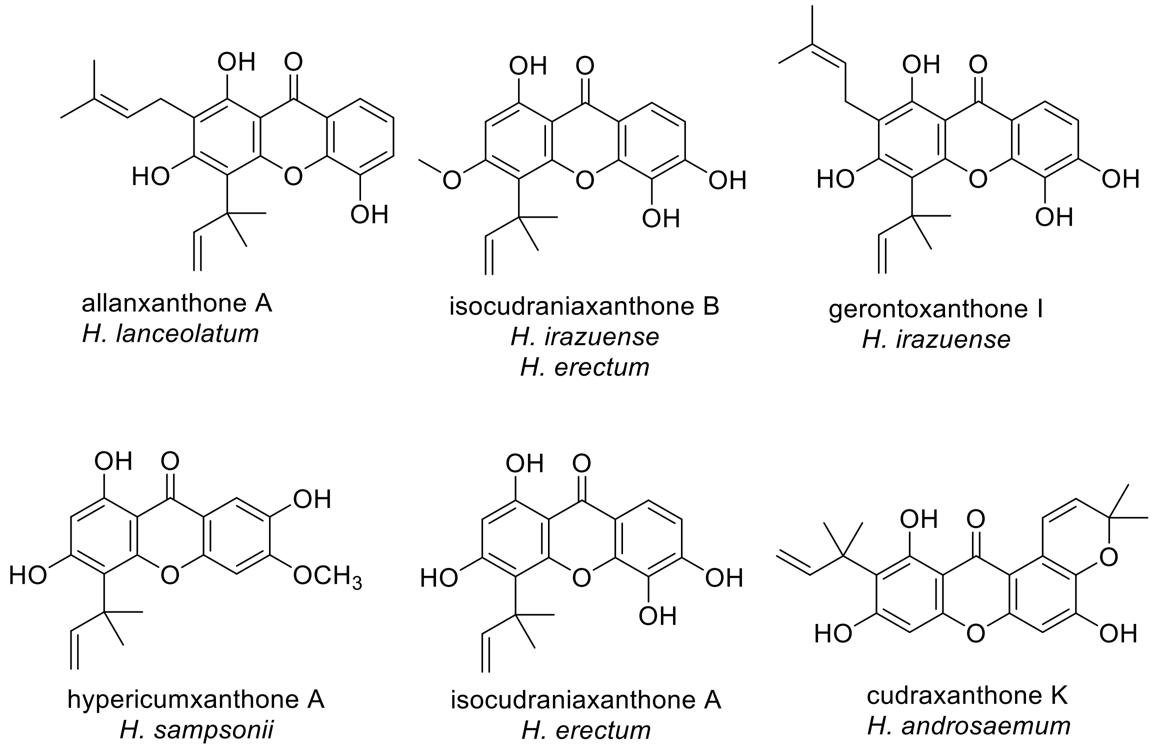


**Figure S20.** Examples of reverse-prenylated xanthones previously reported from various *Hypericum* species.

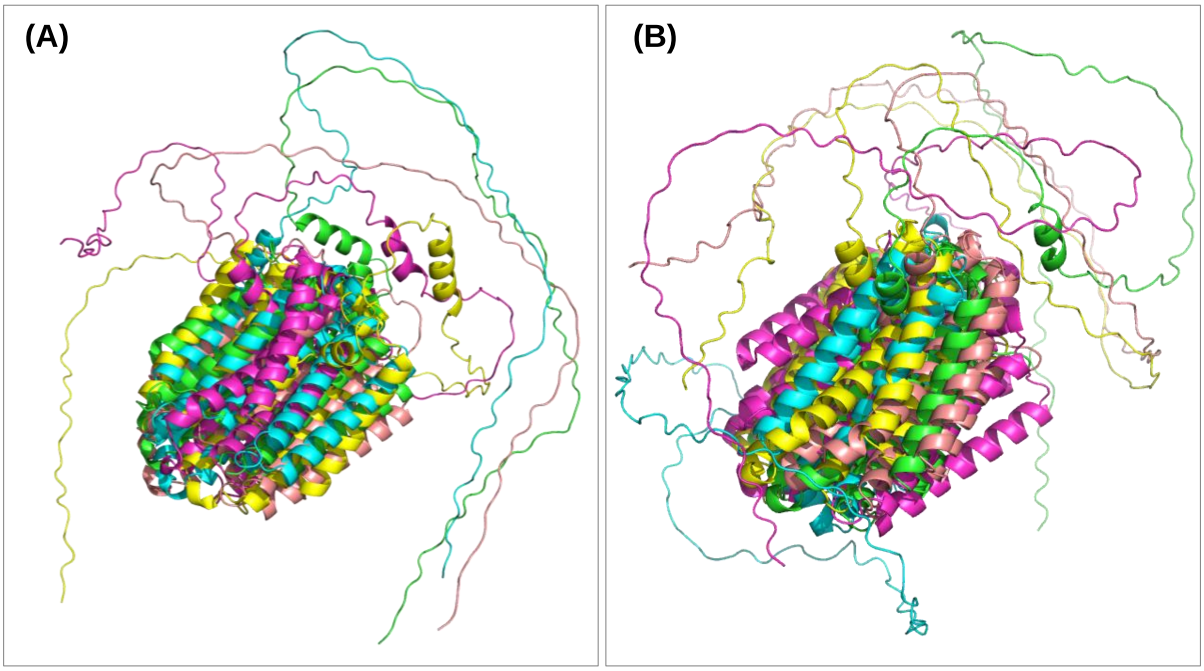


**Figure S21.** Multiple structural alignment of 3D models for (A) *Hp*RPThxa and (B) *Hs*RPThxa built using AlphaFold2. The models are shown as cartoon representations in different colors (model 1 in cyan, model 2 in green, model 3 in pink, model 4 in yellow, and model 5 in orange).

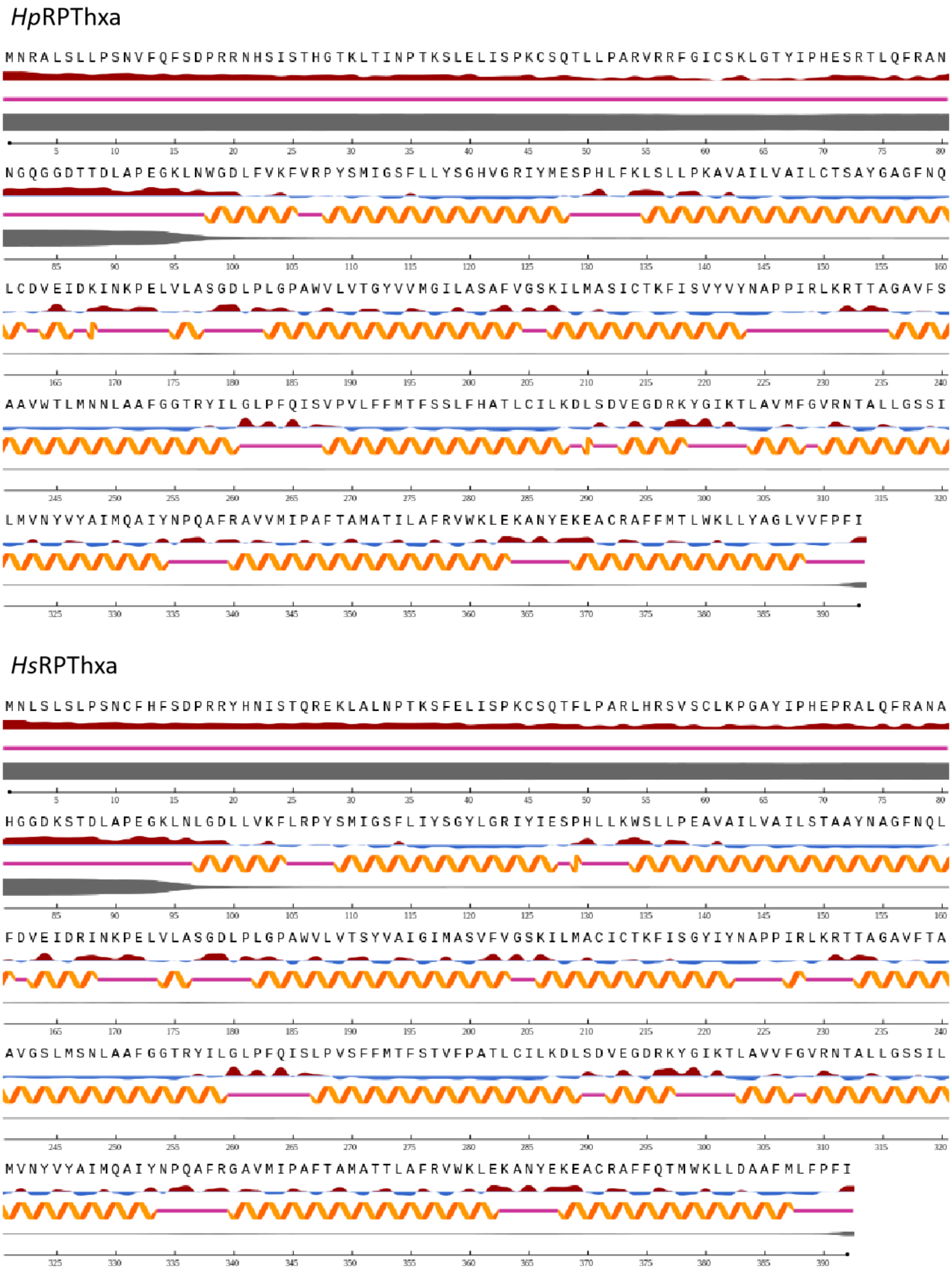


**Figure S22.** Q3 secondary structure prediction for *Hp*RPThxa and *Hs*RPThxa sequences. Predictions were performed using the NetSurfP-3.0 server with default parameters(Høie *et al.*, 2022). The relative surface accessibility is indicated under each residue (red, exposed; blue, buried).

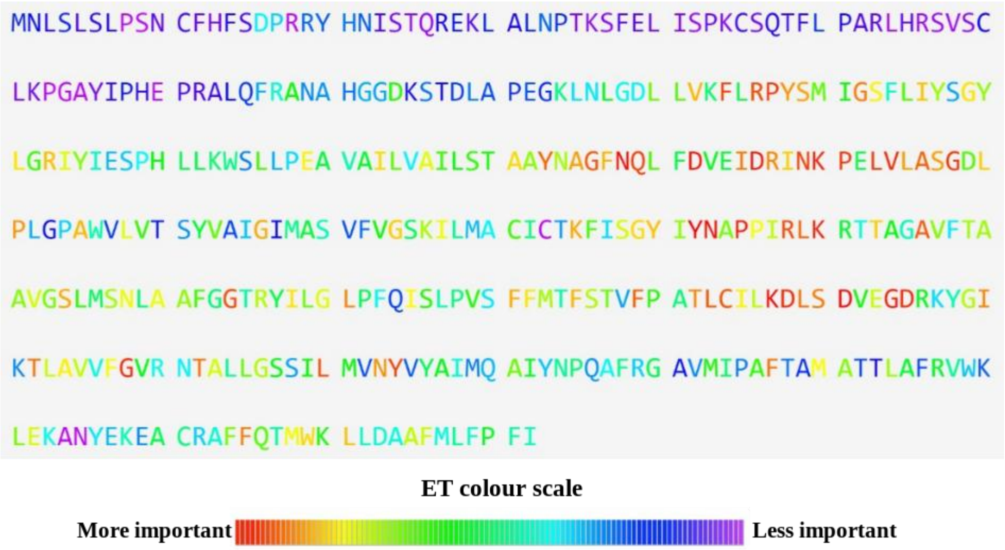


**Figure S23.** Estimation of biological importance of *Hs*RPThxa amino acid residues using the Evolutionary Trace Server with default parameters (Lichtarge *et al.*, 1996, Mihalek *et al.*, 2004, Lua *et al.*, 2015). Potentially important catalytic residues and a low degree of evolutionary conservation of the putative N-terminal leader sequence are found.

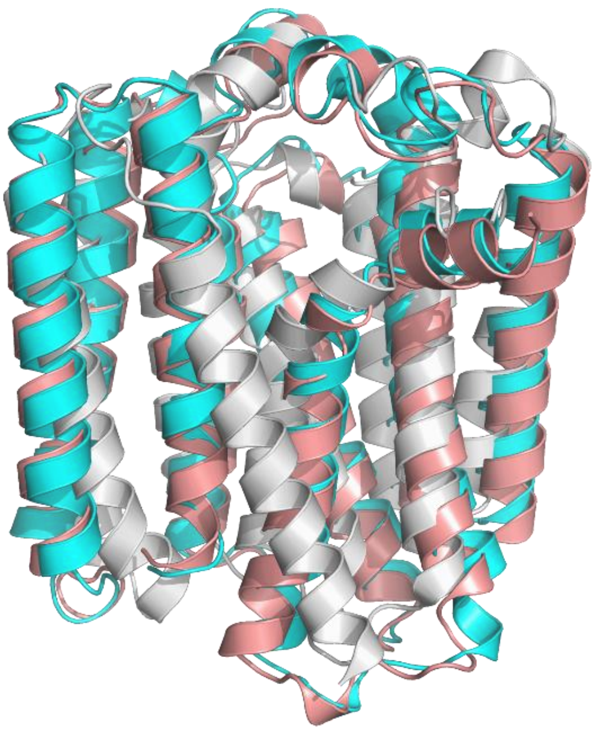

**Figure S24.** Alignment of *Hp*RPThxa (cyan) and *Hs*RPThxa (orange) models with the crystal structure of UBIAD1 (white, PDB ID: 4TQ5), specifying a global RMSD of 4.4 Å. Alignment was performed in YASARA 23.5.19 (Krieger *et al.*, 2002).

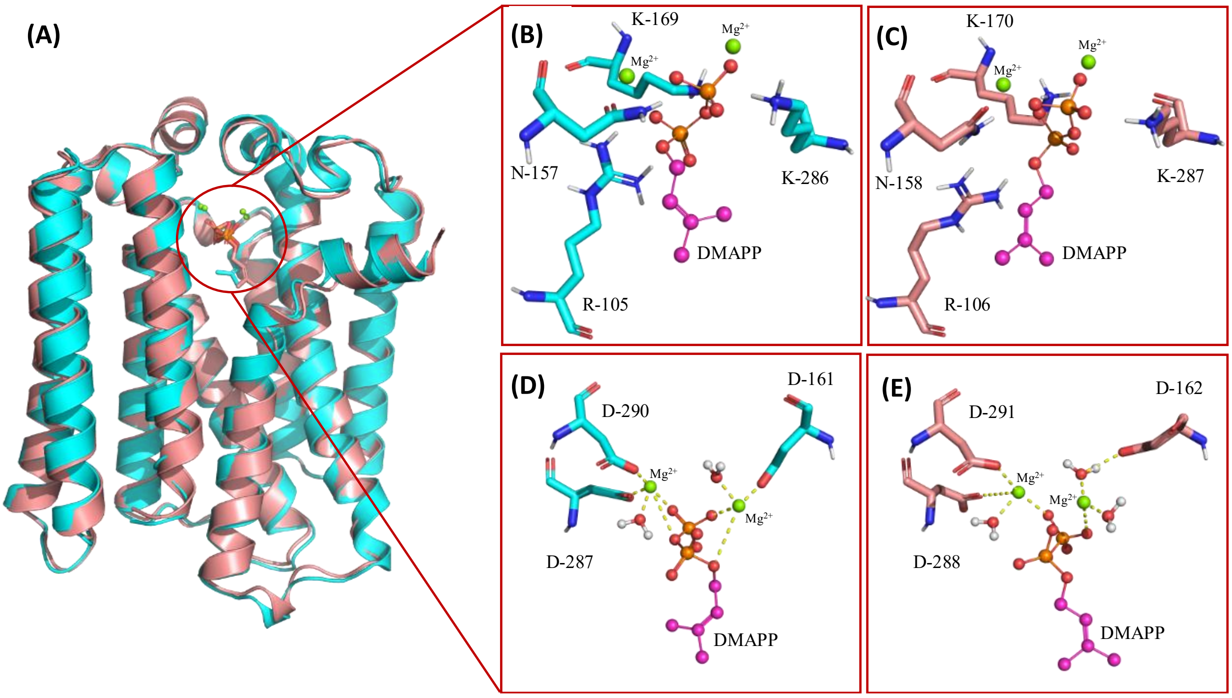


**Figure S25.** Holoenzyme models of *Hs*RPThxa and *Hp*RPThxa showing the interaction with magnesium ions and DMAPP. (A) Overlayed final models of *Hs*RPThxa and *Hp*RPThxa with coordinated $Mg^{2+}$ ions (spheres) and bound DMAPP (sticks). Binding profile of DMAPP (in balls and lines, pink) with the active site residues (in balls and sticks, cyan) rendered in PyMOL2 for (B) *Hp*RPThxa and (C) *Hs*RPThxa. $Mg^{2+}$ (green spheres) binding residues (in sticks) along with the conserved water molecules and DMAPP (in balls and lines, pink) for (D) *Hp*RPThxa and (E) *Hs*RPThxa.

## Supplemental Tables

**Table S1.** Expression data for candidate aPTs from *H. sampsonii* (*H.s.*) transcriptomes and corresponding FPKM values for homologous aPTs from *H. perforatum* (*H.p.*). RNA-seq, providing cross-species expression insights. AA, mature flowers; AB, whole flower buds; AC, whole mature flowers; AD, mature flower petals; AE, pistils, stamens and sepals of flower buds; AF, flower bud petals; AI, middle aged leaves; AJ, old leaves; AK, young leaves, AL, young leaves with light and dark glands; AM, old leaves with light glands; AN, young leaves with light and dark glands; AO, old leaves with light and dark glands; AQ, middle aged part of the roots; AR, oldest part of the roots; AS, dark glands of flower petals; AT, flower petals.

| ***H.s.* transcript** | **Shoot Reads** | **Shoot TPM** | **Root Reads** | **Root TPM** | **Log2FC** | ***H.p.* homolog** | **% Ident.** | **AA** | **AB** | **AC** | **AD** | **AE** | **AF** | **AI** | **AJ** | **AK** | **AL** | **AM** | **AN** | **AO** | **AQ** | **AR** | **AS** | **AT** |
|---|---|---|---|---|---|---|---|---|---|---|---|---|---|---|---|---|---|---|---|---|---|---|---|---|
| DN4386_c0_g3 | 1 | 0.02 | 1175 | 31.30 | 9.20 | hpa_locus_53537 | 86.32 | 0.0 | 0.0 | 0.0 | 0.0 | 0.0 | 0.0 | 2.8 | 2.8 | 0.0 | 0.0 | 0.0 | 3.9 | 0.0 | 9.9 | 9.6 | 0.0 | 0.0 |
| DN4386_c0_g2 | 0 | 0.00 | 198 | 7.54 | 7.64 | hpa_locus_78275 | 85.49 | 0.0 | 0.0 | 0.0 | 0.0 | 0.0 | 0.0 | 0.0 | 0.0 | 0.0 | 0.0 | 0.0 | 0.0 | 0.0 | 7.4 | 3.7 | 0.0 | 0.0 |
| DN5546_c0_g1 | 3 | 0.06 | 646 | 19.39 | 7.34 | - | - | - | - | - | - | - | - | - | - | - | - | - | - | - | - | - | - | - |
| DN38249_c0_g1 | 2 | 0.04 | 126 | 2.53 | 5.40 | - | - | - | - | - | - | - | - | - | - | - | - | - | - | - | - | - | - | - |
| DN8613_c0_g2 | 3 | 0.07 | 120 | 2.85 | 4.92 | - | - | - | - | - | - | - | - | - | - | - | - | - | - | - | - | - | - | - |
| DN19237_c0_g2 | 13 | 0.30 | 222 | 4.99 | 3.99 | hpa_locus_47111 | 91.43 | 0.0 | 0.0 | 0.0 | 0.0 | 0.0 | 0.0 | 0.0 | 0.0 | 0.0 | 0.0 | 10.1 | 0.0 | 6.0 | 0.0 | 0.0 | 0.0 | 0.0 |
| DN23659_c0_g1 | 9 | 0.41 | 128 | 5.85 | 3.69 | hpa_locus_39634 | 87.78 | 0.0 | 0.0 | 0.8 | 1.0 | 0.0 | 0.0 | 0.0 | 0.0 | 0.0 | 0.0 | 0.0 | 0.0 | 0.0 | 2.2 | 2.1 | 0.0 | 2.4 |
| DN19237_c0_g1 | 41 | 0.87 | 374 | 9.62 | 3.16 | hpa_locus_9259 | 75.30 | 55.5 | 47.0 | 43.8 | 0.0 | 22.4 | 11.4 | 1.3 | 0.7 | 4.0 | 1.3 | 1.4 | 1.7 | 2.5 | 0.0 | 0.0 | 0.0 | 0.6 |
| DN2159_c0_g1 | 1995 | 23.52 | 15148 | 175.82 | 2.92 | hpa_locus_27262 | 75.78 | 0.0 | 21.1 | 0.0 | 0.0 | 14.6 | 0.0 | 0.0 | 0.0 | 0.0 | 4.7 | 0.0 | 3.5 | 0.0 | 12.9 | 14.3 | 0.0 | 0.0 |
| DN4386_c0_g1 | 1433 | 25.60 | 8866 | 153.76 | 2.63 | hpa_locus_78275 | 77.72 | 0.0 | 0.0 | 0.0 | 0.0 | 0.0 | 0.0 | 0.0 | 0.0 | 0.0 | 0.0 | 0.0 | 0.0 | 0.0 | 7.4 | 3.7 | 0.0 | 0.0 |
| DN2020_c0_g1 | 669 | 20.41 | 1539 | 45.03 | 1.20 | hpa_locus_4472 | 71.12 | 362.6 | 271.8 | 263.7 | 2.9 | 63.8 | 89.2 | 13.0 | 5.6 | 33.1 | 10.2 | 6.7 | 10.4 | 17.7 | 1.2 | 1.5 | 1.7 | 2.8 |
| DN10571_c0_g1 | 172 | 4.75 | 345 | 14.38 | 1.00 | hpa_locus_15746 | 64.78 | 270.3 | 186.7 | 211.1 | 46.7 | 72.0 | 62.8 | 9.7 | 5.3 | 23.2 | 9.2 | 4.4 | 9.7 | 10.8 | 1.7 | 3.0 | 14.6 | 52.2 |
| DN10571_c0_g1 | 172 | 4.75 | 345 | 14.38 | 1.00 | hpa_locus_4472 | 88.04 | 362.6 | 271.8 | 263.7 | 2.9 | 63.8 | 89.2 | 13.0 | 5.6 | 33.1 | 10.2 | 6.7 | 10.4 | 17.7 | 1.2 | 1.5 | 1.7 | 2.8 |
| DN3075_c0_g1 | 27580 | 456.34 | 44071 | 646.92 | 0.68 | hpa_locus_27262 | 86.34 | 0.00 | 21.1 | 0.0 | 0.0 | 14.6 | 0.0 | 0.0 | 0.0 | 0.0 | 4.7 | 0.0 | 3.5 | 0.0 | 12.9 | 14.3 | 0.0 | 0.0 |
| DN2948_c0_g1 | 402 | 7.59 | 356 | 4.63 | -0.65 | hpa_locus_470 | 64.46 | 334.5 | 283.5 | 249.9 | 25.2 | 48.3 | 84.4 | 15.1 | 7.5 | 47.3 | 14.9 | 14.7 | 11.3 | 19.4 | 0.0 | 0.7 | 5.1 | 28.3 |
| DN2948_c0_g2 | 472 | 10.15 | 156 | 4.11 | -1.45 | hpa_locus_470 | 68.07 | 334.5 | 283.5 | 249.9 | 25.2 | 48.3 | 84.4 | 15.1 | 7.5 | 47.3 | 14.9 | 14.7 | 11.3 | 19.4 | 0.0 | 0.7 | 5.1 | 28.3 |
| DN263_c1_g1 | 561 | 9.47 | 141 | 2.34 | -2.00 | hpa_locus_8393 | 95.34 | 38.6 | 37.4 | 31.5 | 17.2 | 32.0 | 50.0 | 66.5 | 61.9 | 80.3 | 63.4 | 96.2 | 62.5 | 125.4 | 33.2 | 26.7 | 12.9 | 23.0 |

**Table S2.** Kinetic parameters of *Hp*RPThxa, *Hs*RPThxa and *Hs*RPThxa-tr.

| Enzyme | Product | Acceptor | | Donor | |
|---|---|---|---|---|---|
| | | Apparent $K_M$ (µM) | Apparent $V_{max}$ (pmol/s) | Apparent $K_M$ (µM) | Apparent $V_{max}$ (pmol/s) |
| *Hp*RPThxa | Hyperixanthone A | 45.97 ± 4.43 | 1.33 ± 0.03 | 259.99 ± 22.88 | 2.11 ± 0.09 |
| *Hp*RPThxa | Allanxanthone C | 98.84 ±14.52 | 0.20 ± 0.01 | n.a. | n.a. |
| *Hs*RPThxa | Hyperixanthone A | 52.40 ± 4.16 | 3.26 ± 0.09 | 102.21 ± 6.35 | 3.37 ± 0.07 |
| *Hs*RPThxa | Allanxanthone C | 34.626 ± 2.947 | 0.567 ± 0.014 | 299.61 ± 62.46 | 1.203 ± 0.128 |
| *Hs*RPThxa-tr | Hyperixanthone A | 31.33 ± 4.05 | 1.24 ± 0.05 | 30.94 ± 2.58 | 1.38 ± 0.03 |
| *Hs*RPThxa-tr | Allanxanthone C | 14.48 ± 1.39 | 0.140 ± 0.003 | 110.84 ± 6.90 | 0.156 ± 0.003 |

n.a., kinetic parameters could not be calculated due to poor fitting.

**Table S3.** List of the selected residues for *in silico* site saturated mutagenesis.

| Residue | Substrate | Distance (Å) | Energy (kcal/mol) | Type of interaction* |
|---|---|---|---|---|
| Arg106 | DMA | 3.9 | -0.19 | DH |
| Ser109 | PAT | 3.52 | -0.66 | D |
| Ser109 | DMA | 3.99 | -2.52 | D |
| Met110 | PAT | 4.34 | 0.14 | D |
| Ser113 | PAT | 3.85 | -0.41 | D |
| Ile116 | PAT | 4.22 | -0.46 | D |
| Ala151 | PAT | 4.09 | -0.53 | D |
| Asn154 | PAT | 3.84 | -1.67 | D |
| Ala155 | DMA | 4.24 | -0.88 | D |
| Asn158 | DMA | 3.91 | -5.2 | DH |
| Lys170 | DMA | 3.54 | -21.54 | DIH |
| Lys215 | PAT | 3.94 | -7.29 | DH |
| Ser218 | PAT | 3.95 | 0.34 | D |
| Tyr222 | PAT | 4.18 | 0.09 | D |
| Tyr222 | DMA | 3.69 | -5.13 | DH |
| Ala236 | PAT | 4.44 | 0 | D |
| Thr239 | PAT | 3.96 | -0.61 | D |
| Ala240 | PAT | 4.06 | -1.47 | DH |
| Gly243 | PAT | 3.94 | -2.37 | D |
| Ser244 | PAT | 3.96 | -1.94 | D |
| Ser247 | PAT | 4.05 | -0.28 | D |
| Pro280 | PAT | 3.77 | -2.19 | D |
| Cys284 | PAT | 4.06 | -0.65 | D |
| Lys287 | PAT | 3.63 | 0.46 | D |
| Lys287 | DMA | 3.92 | -23.97 | DIH |
| Asp291 | DMA | 4.46 | 0 | D |
| Phe375 | DMA | 4.22 | -0.1 | D |
| Trp379 | PAT | 4.32 | -0.13 | D |
| Trp379 | DMA | 3.88 | -1.18 | D |

*Interaction types The interaction types: D, distance; H; hydrogen bond; I, Ionic

**Table S4.** Changes in stability and affinity of the selected mutants from *in silico* site saturated mutagenesis.

| Mutation | Affinity | Δ Affinity | Stability | Δ Stability |
|---|---|---|---|---|
| S109S | -11.032 | 0.000 | -1109.544 | 0.000 |
| S109A | -11.012 | 0.020 | -1109.071 | 0.474 |
| N154N | -11.038 | 0.000 | -1104.377 | 0.000 |
| N154W | -10.834 | 0.204 | -1102.876 | 1.501 |
| N158N | -11.339 | 0.000 | -1105.051 | 0.000 |
| N158Y | -11.293 | 0.046 | -1104.073 | 0.978 |

**Table S5.** Accession numbers of the aPT sequences used to construct the phylogenetic tree.

| aPT name | Plant species | Accession number |
|---|---|---|
| | **Amaryllidaceae** | |
| ApVTE2-1 | *Allium porrum* | ABB70124.1 |
| | **Apiaceae** | |
| PcPT | *Petroselinum crispum* | BAO31627.1 |
| PsPT1 | *Pastinaca sativa* | AJW31563.1 |
| PsPT2 | | AJW31564.1 |
| AkPT1 | *Angelica keiskei* | BCH36133.1 |
| | **Boraginaceae** | |
| AePGT | *Arnebia euchroma* | ABD59796.2 |
| AePGT4 | | ANC67957.1 |
| AePGT6 | | ANC67959.1 |
| LePGT1 | *Lithospermum erythrorhizon* | BAB84122.1 |
| LePGT2 | | BAB84123.1 |
| | **Brassicaceae** | |
| AtPPT1 | *Arabidopsis thaliana* | NP_567688.1 |
| AtVTE2-1 | | AAM10489.1 |
| AtVTE2-2 | | ABB70127.1 |
| | **Cannabaceae** | |
| CsPT1 | *Cannabis sativa* | DAC76711.1 |
| CsPT4 | | DAC76710.1 |
| HlPT-1 | *Humulus lupulus* | BAJ61049.1 |
| HlPT-2 | | AJD80255.1 |
| | **Ericaceae** | |
| RdPT1 | *Rhododendron dauricum* | BBD96134.1 |
| | **Fabaceae** | |
| AhR3'DT-1 | *Arachis hypogaea* | AQM74173.1 |
| AhR3'DT-2 | | AQM74174.1 |
| AhR3'DT-3 | | AQM74175.1 |
| AhR3'DT-4 | | AQM74176.1 |
| AhR4DT-1 | | AQM74172.1 |
| GmVTE2-1 | *Glycine max* | ABB70126.1 |
| GmC4DT | | BAW32575.1 |
| GmG2DT | | BAW32578.1 |
| GmG4DT | | NP_001235990 |
| GmIDT1 | | BAW32576.1 |
| GmIDT2 | | BAW32577.1 |
| GuA6DT | *Glycyrrhiza uralensis* | AIT11912.1 |
| GuILDT | | AMR58303.1 |

**Table S5.** continued.

| aPT name | Plant species | Accession number |
|---|---|---|
| | **Fabaceae** | |
| LaPT1 | *Lupinus albus* | AER35706.1 |
| LaPT2 | | AWK21939.1 |
| LjG6DT | *Lotus japonicus* | ARV85585.1 |
| PcM4DT | *Psoralea corylifolium* | AYV64464.1 |
| SfFPT | *Sophora flavescens* | AHA36633.1 |
| SfG6DT | | BAK52291.1 |
| SfiLDT | | BAK52290.1 |
| SfN8DT-1 | | BAG12671.1 |
| SfN8DT-2 | | BAG12673.1 |
| SfN8DT-3 | | BAK52289.1 |
| | **Hypericaceae** | |
| HcPT | *Hypericum calycinum* | ALD84371.1 |
| HcPT8px | | AZK16226.1 |
| HcPTpat | | AZK16227.1 |
| HsPT8px | *Hypericum sampsonii* | AZK16224.1 |
| HsPTpat | | AZK16225.1 |
| HsCPTa | | WLY76630.1 |
| HsCPTb | | WLY76631.1 |
| HpPT4px-v1 | *Hypericum perforatum* | OP558033 |
| HpPT4px-v2 | | OP558034 |
| HpCPTa1 | | WXW88711.1 |
| | **Moraceae** | |
| CtIDT | *Cudrania tricuspidata* | AJD80983.1 |
| MaIDT | *Morus alba* | AJD80982.1 |
| MaOGT | | AXN57307.1 |
| FcPT1a | *Ficus carica* | BBC82715.1 |
| | **Polaceae** | |
| HvHGGT | *Hordeum vulgare* | AAP43911.1 |
| OsHGGT | *Oryza sativa* | AAP43913.1 |
| OsPPT1 | | BAE96574.1 |
| OsVTE2-2 | | BAT02293.1 |
| TaHGGT | *Triticum aestivum* | AAP43912.1 |
| TaVTE2-1 | | ABB70123.1 |
| ZmVTE2-1 | *Zea mays* | ACG45339.1 |
| | **Rutaceae** | |
| Cl-PT1a | *Citrus limon* | BAP27988.1 |
| CpPT1 | *Citrus pardisi* | BCH36128.1 |
| CpPT2 | | BCH36129.1 |
| CpPT3 | | BCH36130.1 |
| CmiPT1a | *Citrus micrantha* | BCH36131.1 |
| CmiPT1b | | BCH36132.1 |

**Table S6.** Primer sequences. Restriction sites and modified bases are highlighted in red and green, respectively.

| Primer name | Sequence 5' to 3' |
|---|---|
| **Overexpression primers** | |
| HpRPThxa_F_EcoRI | ATTGAATTCATGAATAGAGCCCTCTCCCTTCTTC |
| HpRPThxa_R_PacI | ATTTTAATTAATTAAATAAAAGGAAATACAACAAGCCCTGCGTAAAG |
| HsRPThxa_F_EcoRI | GCATGAATTCATGAATCTCTCCCTCTCAC |
| HsRPThxa_R_PacI | ATGCTTAATTAATTAAATAAAAGGAAATAACATAAACGC |
| User_HpPThxa F | GGCTTAA[U]ATGAATAGAGCCCTCTCCCTTC |
| User_HpPThxa R | GGTTTAA[U]TTAAATAAAAGGAAATACAACAAGCCCTG |
| User_HsPThxa F | GGCTTAA[U]ATGAATCTCTCCCTCTCACTTC |
| User_truncHsPThxa F | GGCTTAA[U]ATGACTGACCTTGCTCCTGAGG |
| User_HsPThxa R | GGTTTAA[U]TTAAATAAAAGGAAATAACATAAACGCTG |
| User_HsPThxa_CY R | GGTTTAA[U]CCAATAAAAGGAAATAACATAAACGCTG |
| **Mutagenesis primers** | |
| 25trHsRPThxa F | CGAATTCATGCAAAGGGAAAAATTAG |
| 25trHsRPThxa R | CCCTTTGCATGAATTCGAATTTTC |
| HsRPThxa_S109A F | AGGCCTTACgCtATGATCGGATCATTTCTG |
| HsRPThxa_S109A R | TCCGATCATaGcGTAAGGCCTCAAAAATTTCAC |
| HsRPThxa_N154W F | GCAGCTTATtggGCTGGCTTCAACCAAC |
| HsRPThxa_N154W R | AGCCAGCccaATAAGCTGCGGTACTC |
| HsRPThxa_N158Y F | GCTGGCTTCtACCAACTCTTTGATGTCG |
| HsRPThxa_N158Y R | AGAGTTGGTaGAAGCCAGCGTTATAAGC |

**Table S7.** Evaluation of the 3D models predicted by the AlphaFold2 program. Generation of the structural/statistical descriptors QMEAN4 and QMEANDisCo (as Z scores) was done using "QMEAN" – tool of SWISS-MODEL server (https://swissmodel.expasy.org/) (Benkert *et al.*, 2009). The normalized energy Z – score for the models was calculated using the ANOLEA server (http://melolab.org/anolea) (Melo *et al.*, 1997). Geometrical/structural evaluation through Clash and MolProbity scoring was done using the MolProbity server (http://molprobity.biochem.duke.edu/) (Davis *et al.*, 2007). The models used in further analysis are highlighted in yellow.

| Model Name | *Hp*RPThxa | | | | | *Hs*RPThxa | | | | |
|---|---|---|---|---|---|---|---|---|---|---|
| Parameter/ model No. | 1 | 2 | 3 | 4 | 5 | 1 | 2 | 3 | 4 | 5 |
| QMEAN DisCo | 0.47 | 0.49 | 0.48 | 0.48 | 0.49 | 0.47 | 0.47 | 0.47 | 0.47 | 0.47 |
| QMEAN4 | -6.11 | -4.21 | -5.28 | -5.27 | -4.14 | -4.68 | -4.58 | -5.07 | -5.15 | -4.36 |
| Energy Z-score | 4.02 | 3.59 | 3.19 | 4.53 | 3.32 | 4.10 | 4.23 | 4.01 | 4.04 | 4.10 |
| MolProbity (normalized geometry) scores | 2.0 (76$^{th}$ %) | 1.95 (78$^{th}$ %) | 1.74 (88$^{th}$ %) | 1.87 (82$^{nd}$ %) | 1.76 (87$^{th}$ %) | 1.82 (84$^{th}$ %) | 1.95 (78$^{th}$ %) | 1.74 (88$^{th}$ %) | 1.87 (82$^{nd}$ %) | 1.76 (87$^{th}$ %) |
| Clash score | 1.29 (99$^{th}$ %) | 2.42 (99$^{th}$ %) | 2.1 (99$^{th}$ %) | 2.42 (99$^{th}$ %) | 1.45 (99$^{th}$ %) | 1.45 (99$^{th}$ %) | 2.42 (99$^{th}$ %) | 2.1 (99$^{th}$ %) | 2.42 (99$^{th}$ %) | 1.45 (99$^{th}$ %) |

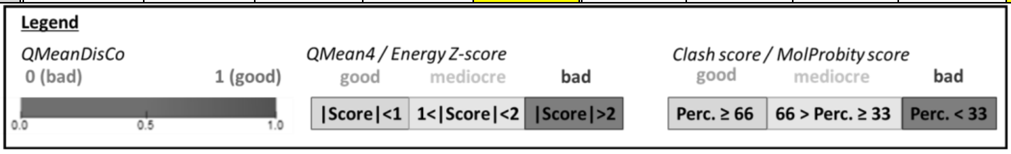

## Supporting References


**Benkert, P., Künzli, M. and Schwede, T.** (2009) QMEAN server for protein model quality estimation. *Nucleic acids research*, **37**, W510-W514.

**Davis, I.W., Leaver-Fay, A., Chen, V.B., Block, J.N., Kapral, G.J., Wang, X., Murray, L.W., Arendall III, W.B., Snoeyink, J. and Richardson, J.S.** (2007) MolProbity: all-atom contacts and structure validation for proteins and nucleic acids. *Nucleic acids research*, **35**, W375-W383.

**Høie, M.H., Kiehl, E.N., Petersen, B., Nielsen, M., Winther, O., Nielsen, H., Hallgren, J. and Marcatili, P.** (2022) NetSurfP-3.0: accurate and fast prediction of protein structural features by protein language models and deep learning. *Nucleic Acids Research*, **50**, W510-W515.

**Krieger, E., Koraimann, G. and Vriend, G.** (2002) Increasing the precision of comparative models with YASARA NOVA—a self‐parameterizing force field. *Proteins: Structure, Function, and Bioinformatics*, **47**, 393-402.

**Lichtarge, O., Bourne, H.R. and Cohen, F.E.** (1996) An evolutionary trace method defines binding surfaces common to protein families. *Journal of Molecular Biology*, **257**, 342-358.

**Lua, R.C., Wilson, S.J., Konecki, D.M., Wilkins, A.D., Venner, E., Morgan, D.H. and Lichtarge, O.** (2015) UET: a database of evolutionarily-predicted functional determinants of protein sequences that cluster as functional sites in protein structures. *Nucleic Acids Research*, **44**, D308-D312.

**Melo, F., Devos, D., Depiereux, E. and Feytmans, E.** (1997) ANOLEA: a www server to assess protein structures. In *Ismb*, pp. 187-190.

**Mihalek, I., Reš, I. and Lichtarge, O.** (2004) A family of evolution–entropy hybrid methods for ranking protein residues by importance. *Journal of Molecular Biology*, **336**, 1265-1282.
